# Assessing Biomass-Based Methanol Production via Electrified Gasification and Solar-Assisted CO_2_ Utilization

**DOI:** 10.1021/acs.iecr.6c00217

**Published:** 2026-06-01

**Authors:** Usman Khan Jadoon, Pullah Bhatnagar, Daniel Flórez-Orrego, Meire Ellen Gorete Ribeiro Domingos, Manuel Rodríguez, François Maréchal

**Affiliations:** † Departamento de Ingeniería Química Industrial y del Medioambiente, Escuela Técnica Superior de Ingenieros Industriales, 16771 Universidad Politécnica de Madrid, 28006 Madrid, Spain; ‡ Industrial Process and Energy Systems Engineering, Ecole Polytechnique Fédérale de Lausanne, EPFL, Sion 1950, Switzerland

## Abstract

This study investigates an electrified biomass-to-methanol synthesis pathway integrated with carbon utilization and renewable energy technologies. Two representative feedstock categories are assessed, namely, lignocellulosic biomass (pine) and agri-food wastes (spent coffee grounds, SCG). Process simulations are conducted in Aspen Plus for four configurations: a fully electrified base case powered by grid electricity, a solar-assisted variant of base case, a methanation-enhanced configuration of base case, and a hybrid system combining both solar electricity and methanation. An advanced process optimization framework is applied to enhance waste heat recovery, power generation, and utility integration, while identifying the minimum energy requirements (MER) of each configuration. Electrification of gasification and reforming processes enhances carbon utilization, achieving carbon efficiencies of 61.6% for lignocellulose and 52.6% for agri-food waste. Methanation further improves carbon recovery to 82.9 and 68.4%, while solar integration increases efficiencies to 87.3 and 73.6%, respectively. Feedstock cost remains the dominant driver of the minimum selling price (MSP), whereas solar integration significantly reduces external utility dependence. Carbon credits for captured biogenic CO_2_ (assumed at 65 €/t) solar-assisted configurations yield methanol MSP of 0.683 €/kg (lignocellulosic biomass) and 0.785 €/kg (agri-food waste), compared with 0.707 and 0.791 €/kg for the corresponding grid-powered cases. Overall, solar-assisted biomass-to-methanol systems show strong potential for techno-economic viability and renewable electricity integration in the Spanish biomass context.

## Graphical abstract

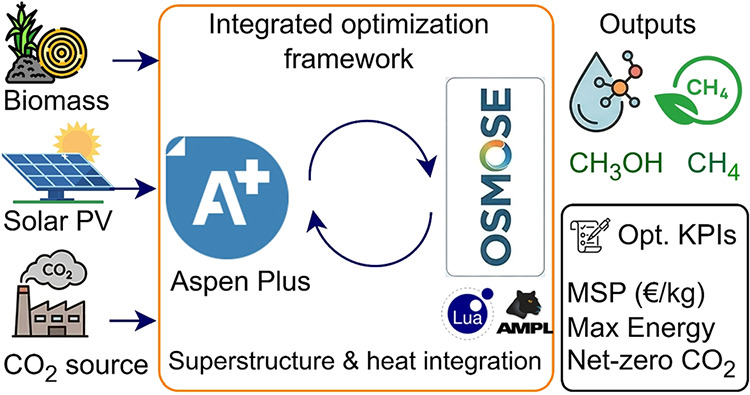

## 1 Introduction

The accelerating depletion of nonrenewable resources, coupled with the escalating risks of climate change and associated ecological impacts, has intensified the global pursuit of sustainable energy alternatives.^1^ In this context, renewable carbon sources capable of displacing fossil feedstocks are receiving increasing attention, particularly for the production of fuels and chemicals that are difficult to electrify directly. Among the available alternatives, waste-derived biomass has emerged as a promising renewable carbon resource, offering the potential to valorize residues while mitigating emissions associated with fossil-based systems.^2^ Rather than relying on assumptions of universal availability, biomass utilization is inherently region-specific and strongly dependent on sustainable resource management to avoid adverse impacts related to land use, biodiversity loss, and competition with food systems. As the only renewable source of biogenic carbon, biomass enables the production of synthetic fuels, platform chemicals, and other biobased materials that are otherwise dependent on fossil inputs. Owing to its diverse feedstock base and localized supply chains, waste biomass can be converted via thermochemical (e.g., gasification, pyrolysis) or biochemical (e.g., fermentation, anaerobic digestion) routes into gaseous, liquid, or solid energy carriers, allowing flexible integration into existing energy and industrial infrastructures.^3^ Biomass is therefore expected to play a significant, though regionally constrained, role in the future renewable energy mix, particularly in sectors requiring carbon-based products.^4^

Among renewable carbon-based fuels, methanol has attracted growing interest due to its versatility as an energy carrier, chemical building block, and drop-in fuel.^5^ Methanol is already widely used in the chemical industry and serves as a key platform chemical for the synthesis of hydrocarbons and oxygenates, positioning it as an important component of emerging circular and low-carbon energy systems.^6,7^ Moreover, its liquid state under ambient conditions offers practical advantages in storage, handling, and distribution compared to gaseous alternatives such as hydrogen, facilitating deployment within existing infrastructure.

Global methanol production has nearly doubled over the past decade, reaching approximately 100 million tonnes per year.^5^ Despite this growth, current production remains predominantly fossil-based, relying mainly on natural gas (≈65%) and coal (≈35%) as primary feedstocks.^5^ These pathways are associated with substantial greenhouse gas emissions. For instance, average direct and indirect fossil CO_2_ emissions from methanol synthesis in Western Europe are approximately 0.76 kg-CO_2_ per kg of methanol, with even higher values when full life-cycle impacts are considered.^8^ In contrast, renewable methanol pathways, particularly those based on biomass, enable the substitution of fossil feedstocks during syngas generation, thereby significantly reducing carbon intensity.^9^ Among these, biomass gasification represents a viable thermochemical route for renewable methanol production, converting lignocellulosic feedstocks into syngas suitable for downstream synthesis.

A comprehensive understanding of the techno-economic performance of biomass-to-methanol systems is essential to evaluate their feasibility and competitiveness in future low-carbon energy scenarios. Over the past two decades, several studies have investigated the efficiency, cost, and integration potential of such processes, providing valuable benchmarks for ongoing research. Hamelinck et al. compared atmospheric and pressurized bubbling fluidized-bed gasifiers with syngas conditioning via water–gas shift and CO_2_ capture (Selexol), reporting energy conversion efficiencies around 55% and production costs of 8–12 $/GJ_HHV_, _MeOH_.^10^ Cifre et al. evaluated hydrogen addition through electrolysis and alternative CO_2_-based routes, finding biomass-derived methanol more favorable (25 to 44% efficiency) than direct CO_2_ conversion, which remained costly and energy-intensive (17–23% efficiency).^11^ Similarly, Clausen et al. explored biomass gasification with H_2_ addition, CO_2_ removal, and biogas reforming, concluding that biomass-based pathways were more efficient (68–72% exergy basis), significantly higher than direct CO_2_ hydrogenation, while noting that electricity prices could account for up to 65% of total synthesis costs.^12^

Peduzzi et al. compared fluidized-bed and entrained-flow gasifiers with CO_2_ removal using monoethanolamine, reporting efficiencies of 43–51% and lower costs at larger scales.^13^ Similarly, Giuliano et al. optimized CO conversion and CO_2_ removal in atmospheric gasification with Selexol, achieving a cost of 0.567 €_2020_/kg-MeOH at 40% CO conversion and 95% CO_2_ removal.^14^ More recently, Domingos et al. investigated the black liquor gasification and conditioning syngas by water–gas shift and CO_2_ capturing (Selexol), reporting exergy efficiency of conventional and integrated processes of 40% and 45%, respectively.^15^

Although previous studies have provided valuable insights into methanol production costs, carbon efficiencies, and system-level optimization, important gaps remain in understanding the combined effects of full-process electrification and renewable electricity integration in biomass-to-methanol systems. While partial electrification has been explored in earlier work, the implications of fully electrifying key process units have remained insufficiently addressed. The integration of renewable electricity, particularly from solar and wind energy technologies, offers opportunities to reduce the reliance on volatile grid electricity prices, improve operational flexibility, and lower overall carbon intensity. Moreover, the direct electrification of major thermal units has received limited attention in process simulations and techno-economic assessments to date. Compared with combustion-based heating, direct electrification provides a controllable and locally emission-free heat supply, making it an attractive option for low-carbon process design.^16^

Recent studies have started to explore the potential of direct and hybrid electrification approaches within thermochemical conversion pathways. Putta et al. demonstrated that combining electrically heated gasification with hydrogen addition improves overall system performance, with an optimal heat input of 37–39% of the biomass energy input, minimizing operating costs.^17^ Similarly, Butera et al. investigated electric heating applied to both tar reforming and gasification, highlighting efficiency improvements and process flexibility.^18^ Melin et al. reported that replacing an oxygen-based allothermal reformer with an electrically heated configuration increased overall energy efficiency from 53.6 to 57.3%, while slightly reducing methanol production costs.^19^ Beyond methanol synthesis, Mesfun et al. examined direct and indirect electrification applied to hydrothermal liquefaction, fast pyrolysis, and ethanol production from sawdust, underscoring the broad applicability of electrification in biomass conversion technologies.^20^

Biomass gasification enables the conversion of lignocellulosic feedstocks into methanol with a reduced carbon footprint; however, conventional gasifiers and tar reformers typically rely on partial combustion for heat supply, which lowers the carbon efficiency and generates additional CO_2_ emissions. To address these limitations, electrification of gasification and reforming units through resistive (Joule) heating has emerged as an alternative to combustion-based heat provisions. This study investigates an integrated biomass-to-methanol system with electrified gasification and tar reforming, considering two waste-derived feedstocks (lignocellulosic and agri-food residues) and four process configurations: (i) an electrified biomass-to-methanol route, (ii) a solar-assisted variant of the first case configuration, (iii) electrified methanol synthesis with auxiliary methanation for synthetic natural gas coproduction, and (iv) a hybrid solar-assisted configuration with methanation. Each configuration is assessed using detailed process modeling and techno-economic analysis, with emphasis on energy integration, carbon utilization, and cost performance. The results provide insights into the combined role of electrification and renewable electricity integration in advancing low-carbon methanol production.

## 2 Methodology

The biomass-to-methanol system investigated in this study integrates thermochemical biomass conversion with renewable electricity utilization and process heat integration. The overall framework combines detailed process simulation with system-level energy optimization and techno-economic analysis. The thermochemical conversion pathway is modeled in Aspen Plus, where individual unit operations, including biomass drying, gasification, gas cleaning, water–gas shift reaction, carbon dioxide separation, and methanol synthesis, are simulated to obtain detailed mass and energy balances. The Aspen Plus results are subsequently integrated with the OSMOSE energy integration framework, which performs systematic heat recovery and utility optimization through pinch-based methods and mixed-integer linear programming (MILP).

Four process configurations are evaluated to investigate the impact of process electrification and renewable hydrogen integration on the system performance. These scenarios compare conventional and electrified gasification concepts and evaluate the potential of using renewable hydrogen produced via solar-powered electrolysis to upgrade captured CO_2_ streams. The integrated modeling framework, therefore, enables the simultaneous evaluation of process performance, energy integration, and techno-economic indicators, providing a comprehensive assessment of the proposed biomass-to-methanol pathways.

### 2.1 Process Configurations and System Boundaries

To assess the influence of different technological configurations on the cost of methanol production, we defined four process scenarios and evaluated them. Each case builds upon the base biomass-to-methanol pathway with successive integration of renewable energy and carbon utilization technologies. To clarify the overall system boundaries, a simplified block flow diagram of the four process scenarios is presented in Figure 1.

**Figure 1:**
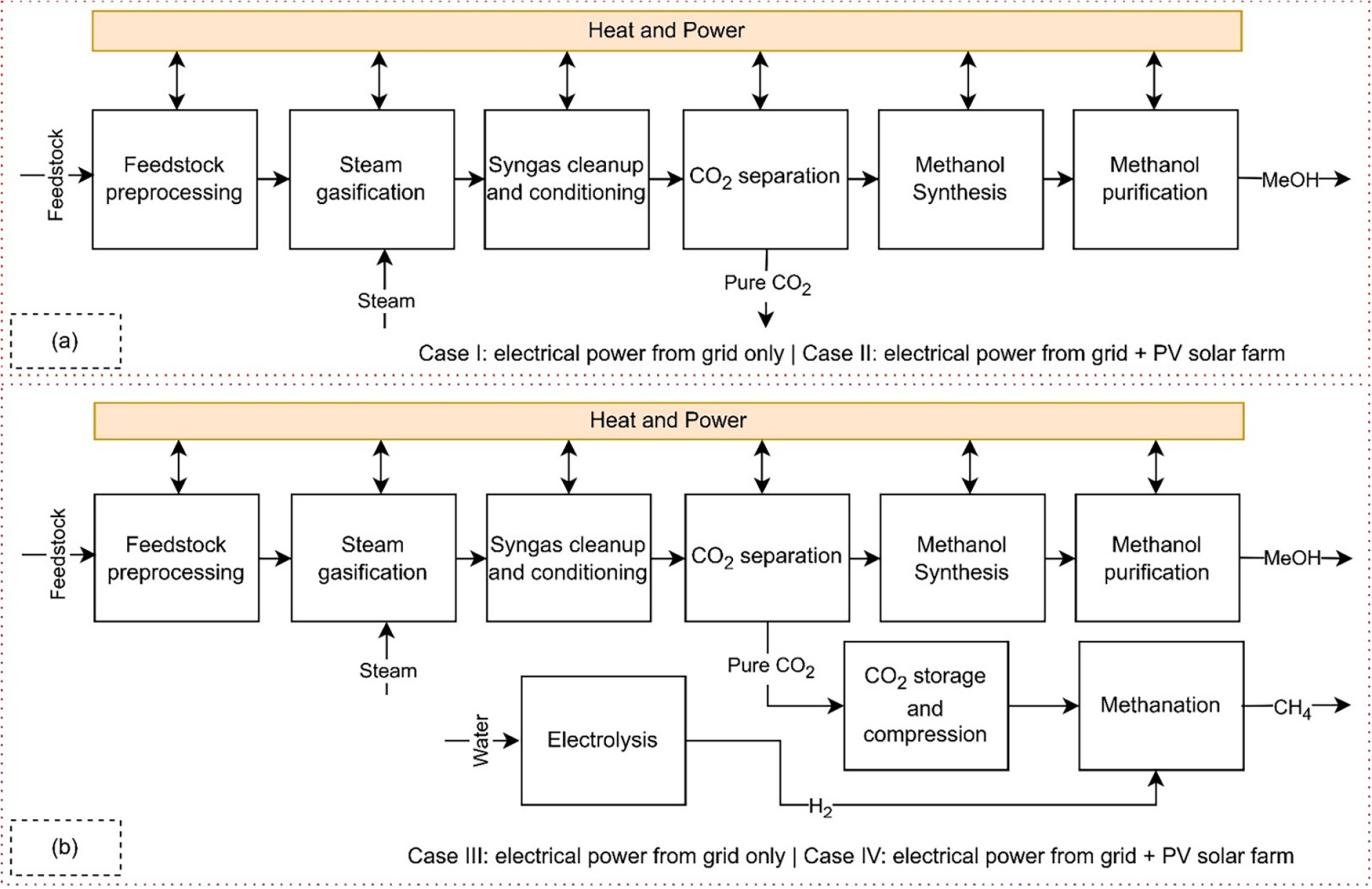
Process framework and system boundaries for the four biomass-to-methanol production scenarios. (a) Biomass-based methanol synthesis with electricity supplied by the grid (Case I) and from a hybrid grid-PV configuration (Case II). (b) Biomass-based methanol synthesis with integrated water electrolysis and CO_2_ methanation for the coproduction of methane, with electricity supplied from the grid only (Case III) and from a hybrid grid–PV configuration (Case IV).

Case I–Reference electrified biomass-to-methanol: This reference configuration considers methanol production from biomass gasification with electrified heat supply, followed by syngas conditioning and methanol synthesis. The complete process, from feedstock conversion to product purification, is modeled and evaluated through techno-economic analysis.

Case II–Solar-assisted biomass-based methanol synthesis: In this case, the plant’s electricity demand is also supplied by solar power, reflecting the integration of renewable electricity potential from the Spanish geographical context. The objective is to quantify the impact of solar integration on methanol production cost.

Case III–Biomass-based methanol synthesis with auxiliary methanation process: CO_2_ captured during syngas conditioning is combined with hydrogen produced via water electrolysis (alkaline electrolyzer). CO_2_ and H_2_ are converted to CH_4_ through a methanation step, providing an additional marketable product alongside methanol.

Case IV–Biomass-based and solar-assisted methanol synthesis with auxiliary methanation process: This configuration combines the features from Cases II and III, with the methanation, and the electricity demand is supplied by solar power as well. This case explores the joint economic benefits of renewable power integration and added product flexibility.

### 2.2 Biomass Cost and Characterization

Biomass resources available in Spain encompass a wide range of agricultural, forestry, and agro-industrial residues. In the present study, the analysis focuses on two representative waste-derived feedstocks: lignocellulosic forestry residues (pine) and agri-food waste in the form of spent coffee grounds (SCG). These feedstocks were selected because of their relevance in the Spanish context and their suitability for thermochemical conversion.

According to data reported by AVEBIOM, the Spanish Biomass Association, the price of wood chips derived from forestry and agricultural residues reached approximately 148.04 €/t per 100 km transport distance in 2024.^21^ Higher costs have been reported for bagged olive pits, reaching up to 252.26 €/t for transport distances of 200 km.^21^ Currently, 57 facilities produce wood chips, and 29 produce olive pits in Spain, with detailed information on plant capacities and locations compiled by AVEBIOM.^22^ In contrast, consistent and publicly available cost data for agri-food waste streams remain limited, as these materials are generated in a highly dispersed manner across cafés, restaurants, and industrial kitchens. For such residues, collection and logistics represent the dominant cost components. A study conducted in Aveiro (Portugal) reported food waste collection costs ranging from 79 to 100 €/t, primarily driven by transportation and handling requirements.^23^ Given the lack of harmonized cost data for agri-food residues in Spain and to ensure methodological consistency across feedstocks, a conservative average cost of 120 €/t was assumed for both lignocellulosic residues and agri-food waste.^24^ In this study, forestry biomass^25^ and agri-food have been selected as feedstock, and their proximate and ultimate analyses are given in Table 1. The compositional data for spent coffee grounds were obtained from the Phyllis2 database (ID #1788).^26^

**Table 1:** Analysis of the Feedstock Waste

parameter	pine (lignocellulosic biomass)	spent coffee grounds (agri-food)
Proximate Analysis (%)		
Moisture	3.3	0
Ashes	1.3	0.3
Volatile matter	77.2	86.40
Fixed carbon	18.3	13.30
Ultimate Analysis (%)		
C	51.5	57
H	5.8	7.6
N	0.3	2.10
S	0	0.10
O	37.8	32.9
LHV (kJ/kg)	17,941	23,620

### 2.3 Process Modeling and Simulation

Figure 2 shows the process flowsheet of the methanol synthesis. Aspen Plus v14.0 software is used to evaluate the mass, energy, and exergy balances for all units, considering the Peng–Robinson equation of state with Boston-Mathias modifications. The perturbed-chain statistical associating fluid theory (PC-SAFT) correlation is used to model the physical absorption of CO_2_ with dimethyl ethers of poly­(ethylene glycol) (DEPG).^27^ Finally, the NRTL-RK property method (nonrandom two liquid activity-coefficient model with Redlich–Kwong equation of state) is used for modeling the distillation section of methanol, since it is suitable for describing the phases present in those systems.

**Figure 2:**
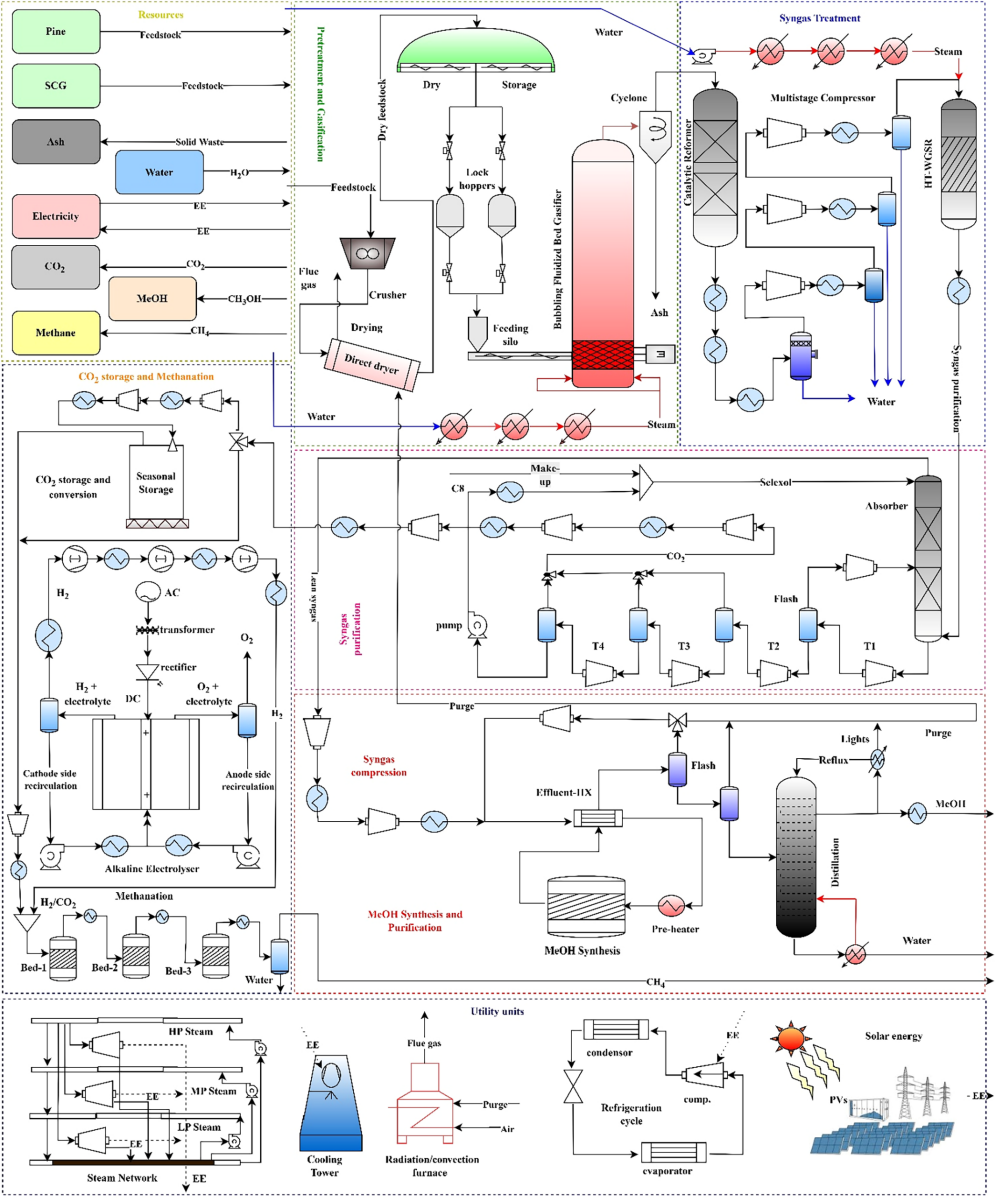
Process flowsheet of methanol synthesis.

#### 2.3.1 Intermediate Storage and Drying

In this study, both woody biomass and agri-food wastes are assumed to undergo an initial intermediate storage phase. The feedstocks are accumulated at a regional storage center, where they are naturally air-dried until reaching a moisture content of roughly 35%.^28^ Agri-food waste is also assumed to arrive at the storage facility with an initial moisture level representative of typical collected material. Following storage, the biomass is transported to the conversion site and dried to the required moisture level for the gasification. The drying process is modeled at 120 °C and atmospheric pressure using a direct-contact rotary dryer. Thermal energy is supplied via direct contact with hot gases from the combustion of the synthesis loop purge gas, as utilized in the agri-food waste scenario. Direct steam injection was not considered for this unit, as introducing steam would increase the partial pressure of water in the drying atmosphere, thereby reducing the driving force for moisture evaporation. The energy requirement is calculated for reducing the incoming moisture content to 10% (wet basis). To quantify this demand, a simplified drying-energy correlation derived from established rotary kiln drying models is employed, following methodologies commonly applied in biomass pretreatment studies.^29,30^

#### 2.3.2 Biomass Gasification

The biomass feedstock undergoes preliminary preparation involving debarking, chipping, and classification into wood chips, during which approximately 10% of the original mass is lost.^31^ The prepared biomass is then fed into a bubbling fluidized-bed gasifier (BFBG), where it is thermochemically converted into syngas, tar, and char. Gasification reactions in the BFBG proceed through a sequence of drying, devolatilization, partial oxidation, and char gasification, leading to a complex mixture of CO, H_2_, CO_2_, CH_4_, and trace hydrocarbons. The reactor configuration and operating conditions were adopted from Yang et al., who achieved stable pine biomass gasification at 800 °C and a steam to biomass ratio of 0.9 with a weight hourly space velocity of 828 kg m^–3^h^–1^.^32^ Under these conditions, the molar flow rates of syngas constituents were determined using the kinetic data as detailed in a previous publication (Jadoon et al.).^33^ However, the prediction of syngas composition was performed using a machine learning (ML) model trained on experimental data sets, as detailed in the same work.^33^ This surrogate ML approach enabled accurate prediction of the outlet syngas composition (H_2_, CO, CO_2_, and CH_4_ % mole fractions).

A key design consideration for the BFBG is the supply of heat required to sustain the endothermic steam gasification reactions. Instead of conventional autothermal operation using partial combustion of syngas or char, this study considers an electrified allothermal configuration. Physically, this is envisioned as a network of electrical resistance heaters (e.g., high-temperature metallic alloys or silicon carbide) housed within protective, highly thermally conductive thermowells or radiant tubes (such as Incoloy or advanced ceramics).^34^ These protective tubes are directly immersed in the bed region of the BFBG. The intense multiphase mixing characteristic of the BFBG ensures exceptionally high bed-to-tube heat transfer coefficients, allowing the required endothermic heat of gasification to be supplied efficiently via conduction and convection.^35^ To ensure operational stability, the skin temperature of these heating tubes must be strictly controlled to remain below the ash fusion temperature of the biomass feedstock, thereby preventing localized sintering or agglomeration of the bed material. The specific thermodynamic electrical energy requirement (0.26 MW/tonne-biomass) was derived from the conceptual design proposed by Klüh et al.^36^ Their analysis demonstrated that the thermal energy demand of the gasifier is supplied through electrical heating of the circulating bed material and/or reactor walls, and surface temperature of the resistive elements remains below the ash fusion temperature of the biomass feedstock. This operational safeguard prevents sintering or agglomeration of bed particles, thereby ensuring stable fluidization.

Alternatively, electrical heating elements may be integrated behind the refractory lining of the gasifier wall, allowing heat transfer to the fluidized suspension through a combination of convective and radiative heat transfer mechanisms.^34^ This electrified configuration enables the gasifier to operate without internal combustion reactions, thereby improving syngas quality and facilitating integration with renewable electricity sources. The BFBG configuration is particularly suitable for such electrified operation because the vigorous particle–gas mixing within the bed results in high effective heat transfer coefficients between the heating surfaces, bed material, and reacting biomass particles. This rapid heat redistribution ensures nearly uniform bed temperatures and allows the externally supplied electrical heat to be efficiently transferred to the endothermic gasification reactions.^36^

The ash is recovered from the gasifier and is safely handled as solid waste. Furthermore, to manage trace sulfur impurities (such as the 0.1 wt % sulfur present in the agri-food waste) and prevent downstream catalyst poisoning in the reformer and water gas shift units, in-bed desulfurization is assumed. A calcium-based sorbent, such as limestone, is cofed with the biomass directly into the BFBG. This captures volatile sulfur as solid calcium sulfide (CaS),^37–39^ which is continuously discharged alongside the ash, ensuring gas purification without the need for supplementary gas-cleaning vessels. The use of calcium-based bed materials in BFBGs enables in situ sulfur capture, thereby reducing sulfur concentrations before the syngas enters downstream catalytic reactors.

#### 2.3.3 Tar Reforming

The raw syngas exiting the BFBG contains not only the volatile components but also undesired hydrocarbons and condensable tars. These heavier species reduce the quality of syngas for downstream methanol synthesis and must be reformed to lighter molecules. In this study, a dedicated tar reformer was incorporated downstream of the gasifier to convert tars and light hydrocarbons primarily into CO and H_2_.^40^ The tar reformer was modeled using an RStoic reactor in Aspen Plus, where the conversion rates of key species, comprising both noncondensable light hydrocarbons (CH_4_, C_2_H_6_, C_2_H_4_) and condensable tar fractions (C_6_H_6_, and C_10_^+^ fractions), were specified according to experimental data reported by Spath et al.^41^ The desired specified conversion efficiencies are listed in Table 2. For example, CH_4_ was targeted at 80% conversion, while higher hydrocarbons such as benzene (C_6_H_6_) and polyaromatics (C_10_^+^) were assigned near-complete conversions.^41^ These values ensure that the syngas achieves a composition suitable for conditioning and the subsequent catalytic synthesis of methanol.

**Table 2:** Conversion Efficiencies of Light Hydrocarbons and Condensable Tars into CO and H_2_

compound	% conversion to CO and H_2_
CH_4_	20%
C_2_H_6_	99%
C_2_H_4_	90%
C_6_H_6_	99%
C10+	99.9%

The primary reaction mechanism governing the reforming process can be generalized as by eq 1

1 $$C_{n}H_{m}+nH_{2}O\to \left(n+m/2\right)H_{2}+n\mathrm{CO}$$

This strongly endothermic reforming reaction requires a continuous heat supply to maintain reactor temperature. Conventionally, such heat is provided through natural gas combustion in external furnaces. However, in this work, the system was fully electrified by supplying the required thermal energy through Joule (resistive) heating. To estimate the electrical power demand of this novel heating configuration, insights were drawn from Lu and Nikrityuk, who developed a multiscale particle-based model of steam methane reforming in electrically heated fixed beds. Their work demonstrated how direct current passing through electrically conductive particles (e.g., nickel) enables volumetric heating of the catalyst bed.^42^ The operating temperature was maintained at 800 °C, consistent with both gasifier outlet conditions and kinetics.

#### 2.3.4 Water Gas Shift Reactor

Following tar reforming, the syngas composition is further conditioned to achieve the appropriate H_2_/CO molar ratio of 2:1, a critical parameter for methanol synthesis. The outlet gas from the tar reformer is cooled to approximately 30 °C in a heat exchanger, compressed to 30 bar, and then directed to the high-temperature (HT) water–gas shift (WGS) section at 300 °C and 30 bar. The WGS reaction was modeled using an RPlug reactor in Aspen Plus operated adiabatically. The kinetic scheme was implemented as a power-law reversible reaction model, with the pre-exponential factor, activation energy, and reaction orders adopted from the kinetic data reported by Hla et al.^43^ In Aspen Plus, the HT-WGS unit was modeled based on the study carried out by Patra et al., ensuring that the plug-flow assumption realistically approximated the experimental data.^44^ For the provision of steam, water is pressurized to 30 bar and then steam generated at 300 °C.

The principal reaction in the HT-WGS unit is given by eq 2

2 $$\mathrm{CO}+H_{2}O⇌{\mathrm{CO}}_{2}+H_{2},\Delta H=-41.2\mathrm{kJ}/\mathrm{mol}$$

This exothermic equilibrium-limited reaction increases the hydrogen fraction in syngas, while reducing CO, thereby improving the stoichiometric number required for methanol synthesis. The overall rate expression employed in the model is given by eq 3

3 $$r=10^{0.659}\exp\left(\frac{-88}{\mathrm{RT}}\right)P_{\mathrm{CO}}^{0.9}P_{H_{2}O}^{0.31}P_{{\mathrm{CO}}_{2}}^{-0.156}P_{H_{2}}^{-0.05}\left(1-\beta \right)$$

where the β is defined in eq 4

4 $$\beta =\frac{P_{{\mathrm{CO}}_{2}}P_{H_{2}}}{KP_{\mathrm{CO}}P_{H_{2}O}}$$

The equilibrium constant (*K*) was determined from the correlation reported by Callaghan with *T* expressed in kelvin.^45^

5 $$\log_{10}K=-2.1497+0.0003855T+\frac{2180.6}{T}$$

#### 2.3.5 CO_2_ Capture and Purification

After the HT-WGS reactor, the syngas is cooled to 30 °C and directed to the gas purification section, where the key objective is the selective capture of CO_2_ to condition the gas for methanol synthesis. The CO_2_ removal unit was modeled as a high-pressure absorption column operating at 30 bar, where the syngas is contacted with DEPG solvent.^15^ In this physical absorption process, CO_2_ is preferentially absorbed into the liquid phase, yielding a CO_2_-rich bottoms stream and a purified syngas overhead.^46^ An important design consideration for methanol synthesis is the stoichiometric number (SN), defined as eq 6

6 $$\mathrm{SN}=\frac{y_{H_{2}}-y_{{\mathrm{CO}}_{2}}}{y_{\mathrm{CO}}+y_{{\mathrm{CO}}_{2}}}$$

For optimal operation of the methanol loop, the SN must be maintained slightly above 2.0.^47^ This ensures a balanced feed composition, maximizes CO_*x*_ conversion, and aligns with commercial practices that favor high CO/CO_2_ ratios for enhancing reaction rates.

The CO_2_ capture model was adapted from a reference study employing Selexol as a physical solvent in an absorber-flashing configuration, where four sequential flash tanks were used for CO_2_ desorption, achieving a reported capture efficiency of nearly 99.99%.^48,49^ In this study, the CO_2_ capture unit was modeled using a Radfrac absorber operating at 30 bar and 30 °C. The absorber section has 20 stages with a total height of 10.97 m, and an implied HETP of 0.61 m. The tower diameter was sized at 3.0 m, corresponding to 60% flooding, with an overall section pressure drop of 0.24 bar. Rich solvent leaving the absorber undergoes four-stage depressurizations at 20, 10, 5, and 1 bar (flash temperature is 25 °C). Power recovery is achieved through staged expansion, while light gases released are recompressed and recycled to the absorber. The model achieved a CO_2_ capture ratio of 99%, with a solvent circulation rate of 71 kg-solvent/kg-CO_2_-captured.

#### 2.3.6 Methanol Synthesis

The methanol synthesis reactor represents one of the most critical units in the overall process design as its performance largely determines plant efficiency, conversion rates, and economic viability. Accurate reactor modeling reinforces the conceptual design, simulation, and eventual scale-up of the process. The reliability of this step is dependent primarily on the kinetic framework implemented in the model.

The Aspen Plus model of methanol synthesis loop was carried out by using the RK-Soave equation of state, which is suitable for nonpolar and weakly polar species such as H_2_, CO, CO_2_, CH_3_OH, and H_2_O under high-pressure conditions and moderate temperatures.^50^ For the distillation section, where polar interactions dominate and hydrogen is absent, the NRTL activity model was applied.^51^ Both approaches are in line with literature recommendations for similar systems, and all binary interaction parameters were taken directly from the Aspen Plus component data bank.

Methanol formation over a Cu/ZnO/Al_2_O_3_ catalyst proceeds through three principal equilibrium reactions given in eqs 7–9

7 $$\begin{array}{ll} \mathrm{CO}+{2H}_{2}⇌{\mathrm{CH}}_{3}\mathrm{OH} & \Delta H^{{}^{\circ}}=-90.77\mathrm{kJ}/\mathrm{mol} \end{array}$$

8 $$\begin{array}{ll} {\mathrm{CO}}_{2}+H_{2}⇌\mathrm{CO}+H_{2}O & \Delta H^{{}^{\circ}}=+41.21\mathrm{kJ}/\mathrm{mol} \end{array}$$

9 $$\begin{array}{ll} {\mathrm{CO}}_{2}+{3H}_{2}⇌{\mathrm{CH}}_{3}\mathrm{OH}+H_{2}O & \Delta H^{{}^{\circ}}=-49.16\mathrm{kJ}/\mathrm{mol} \end{array}$$

Reactions A and C are exothermic and favored by high pressure and low temperature, whereas reaction B (the reverse water–gas shift) is endothermic and promoted at higher temperatures. As a result, methanol yield increases at lower temperatures and elevated pressures. For balanced operation, the SN must be maintained near 2.0. The reactor was modeled using the Langmuir–Hinshelwood–Hougen–Watson kinetic expressions reformulated by Kiss et al., which explicitly account for adsorption phenomena on the catalyst surface.^52^ The reactor configuration and catalyst specification are given in Table 3. The corresponding rate expressions are given in eqs 10–12.^53^ Whereas the generalized rate expressions, kinetic factors, and driving force constants are given in supplementary data in Tables S1 and S2.

**Table 3:** Methanol Synthesis Reactor Configuration and Catalyst Specification

parameter	value
Reactor diameter (m)	3.5
Reactor length (m)	0.04
Number of tubes	980
Catalyst diameter (m)	0.0054
Catalyst density (kg/m^3^)	1180
Bed voidage (-)	0.285
Catalyst shape factor (-)	1
Heat transfer coefficient (W/(m^2^K))	118.44

10 $$r_{{\mathrm{CH}}_{3}\mathrm{OH}}=k_{A}\frac{K_{\mathrm{CO}}\left[f_{\mathrm{CO}}f_{H_{2}}^{3/2}-f_{{\mathrm{CH}}_{3}\mathrm{OH}}/\left(K_{A}\sqrt{f_{H_{2}}}\right)\right]}{\left(1+K_{\mathrm{CO}}f_{\mathrm{CO}}+K_{{\mathrm{CO}}_{2}}f_{{\mathrm{CO}}_{2}}\right)\left[\sqrt{f_{H_{2}}}+\left(K_{H_{2}O}/\sqrt{K_{H}}\right)f_{H_{2}O}\right]}$$

11 $$r_{\mathrm{CO}}=r_{H_{2}O}=k_{B}\frac{K_{{\mathrm{CO}}_{2}}\left[f_{{\mathrm{CO}}_{2}}f_{H_{2}}-f_{H_{2}O}f_{\mathrm{CO}}/K_{B}\right]}{\left(1+K_{\mathrm{CO}}f_{\mathrm{CO}}+K_{{\mathrm{CO}}_{2}}f_{{\mathrm{CO}}_{2}}\right)\left[\sqrt{f_{H_{2}}}+\left(K_{H_{2}O}/\sqrt{K_{H}}\right)f_{H_{2}O}\right]}$$

12 $$r_{{\mathrm{CH}}_{3}\mathrm{OH}}=r_{H_{2}O}=k_{C}\frac{K_{{\mathrm{CO}}_{2}}\left[f_{{\mathrm{CO}}^{\prime }}f_{H_{2}}^{3/2}-f_{H_{2}O}f_{{\mathrm{CH}}_{3}\mathrm{OH}}/\left(K_{C}f_{H_{2}}^{3/2}\right)\right]}{\left(1+K_{\mathrm{CO}}f_{\mathrm{CO}}+K_{{\mathrm{CO}}_{2}}f_{{\mathrm{CO}}_{2}}\right)\left[\sqrt{f_{H_{2}}}+\left(K_{H_{2}O}/\sqrt{K_{H}}\right)f_{H_{2}O}\right]}$$

The methanol synthesis kinetics were validated by reproducing the experimental conditions reported in ref.^52^ The implemented model was tested across process variables representative of industrial practice of 200–300 °C and 50–100 bar. Because the hydrogenation of both CO and CO_2_ involves a net reduction in molar volume, increasing pressure was found to enhance CO conversion to methanol. On the contrary, temperature had a dual effect as per Yang et al., since below 240 °C, CO tends to dominate as the carbon source, and above this threshold, CO_2_ becomes the main contributor for methanol synthesis.^54^ Given that biomass-derived syngas typically contains more CO than CO_2_, operating at moderately low temperatures is advantageous to favor CO hydrogenation. Based on this combined evaluation, an operating point of 210 °C and 90 bar was selected for the synthesis loop, as it provides favorable process conditions between kinetics and thermodynamics while ensuring high methanol yield.

The purified syngas is compressed to 90 bar and fed to the methanol synthesis loop, where it is preheated in a feed–effluent heat exchanger using the hot reactor effluent before entering an isothermal reactor operating at 210 °C and 90 bar. The reactor effluent, consisting of methanol, water, and unconverted reactants, is cooled and subjected to two successive flash separations, first at 45 bar and 30 °C, and then at 3.5 bar, to remove condensable products from the gas phase. Because per-pass conversion of methanol is limited by chemical equilibrium, a recycle loop is necessary. To avoid the accumulation of inert gases, a purge stream is required, which involves a loss of reactants and then a decrease in the production rate. The liquid fraction, containing crude methanol and water, is routed to a distillation column at atmospheric pressure to get methanol with a purity exceeding 99 wt %. The gaseous fraction is partially purged to prevent inert buildup, while the remainder is recycled back to the reactor loop to enhance overall conversion efficiency.

### 2.4 Utility Systems Integration

The integrated chemical plant requires auxiliary energy support for the production of methanol and associated utility operations. Therefore, the selection and configuration of utility systems are critical and strongly dependent on the relative costs associated with the processing plant (Figure 2). The extent of waste heat recovery and reuse is influenced by the specific combination of utilities adopted, which demands an iterative energy integration approach. The identification of the optimal combination of energy resources and technologies, together with their appropriate operating conditions, has become a complex decision process involving numerous feasible configurations.

The main utility systems employed in the process include steam generation, cooling water, and power recovery units, as shown in Figure 2. In particular, the treatment of the purge stream from the methanol synthesis loop can significantly influence overall plant performance. Different purge gas treatment strategies have been reported in the literature, including purification and recycle to the synthesis loop,^55^ direct use as an energy carrier,^56^ or selective CO_2_ recovery.^57^ In this study, for the woody biomass gasification case, the purge gas is combusted to generate electricity, thereby improving energy self-sufficiency and reducing external power demand. In contrast, for agri-food waste used as feedstock, the purge gas is used as a heat source for drying the biomass feed. Meanwhile, a mechanical draft-cooling tower system provides the cold utility balance, which has inlet and outlet temperatures at 40 °C and 25 °C, respectively, and an electricity-to-cooling duty ratio of 0.021 kWh_el_/kWh_th_ is assumed.^58^ Additionally, a set of backpressure and extraction-condensation steam turbines optimally profits from the thermodynamic potential of the waste heat to generate power. The choice of the optimal pressure levels of steam is carried out by inspecting the profile of the grand composite curve of the methanol synthesis.^59^

In addition to internal energy recovery, a solar photovoltaic (PV) farm is incorporated into the system to supply renewable electricity during daylight hours. The PV system is designed using real-time solar irradiance data by a photovoltaic geographical information system corresponding to the site location in Madrid, Spain (latitude 40.261°, longitude −3.803°) with a plane tilt of 35°.^60^ The yearly global horizontal irradiance (GHI) profile is presented in Figure 3, and an average value of 472.8 W/m^2^ was considered for sizing the solar farm. The integration between the PV system and the chemical plant is synchronized within the energy integration framework. During daytime operation, the process utilizes power from both the PV farm and waste heat-recovery systems, minimizing dependence on grid electricity. When the PV generation exceeds the plant’s instantaneous power demand, the surplus electricity is exported to the national grid. Conversely, during nighttime or periods of low solar output, electricity is drawn from the grid to maintain continuous plant operation. This coordinated integration between renewable generation, internal recovery, and grid exchange ensures stable process operation while improving the overall energy efficiency and sustainability of the biomass-to-methanol system.

**Figure 3:**
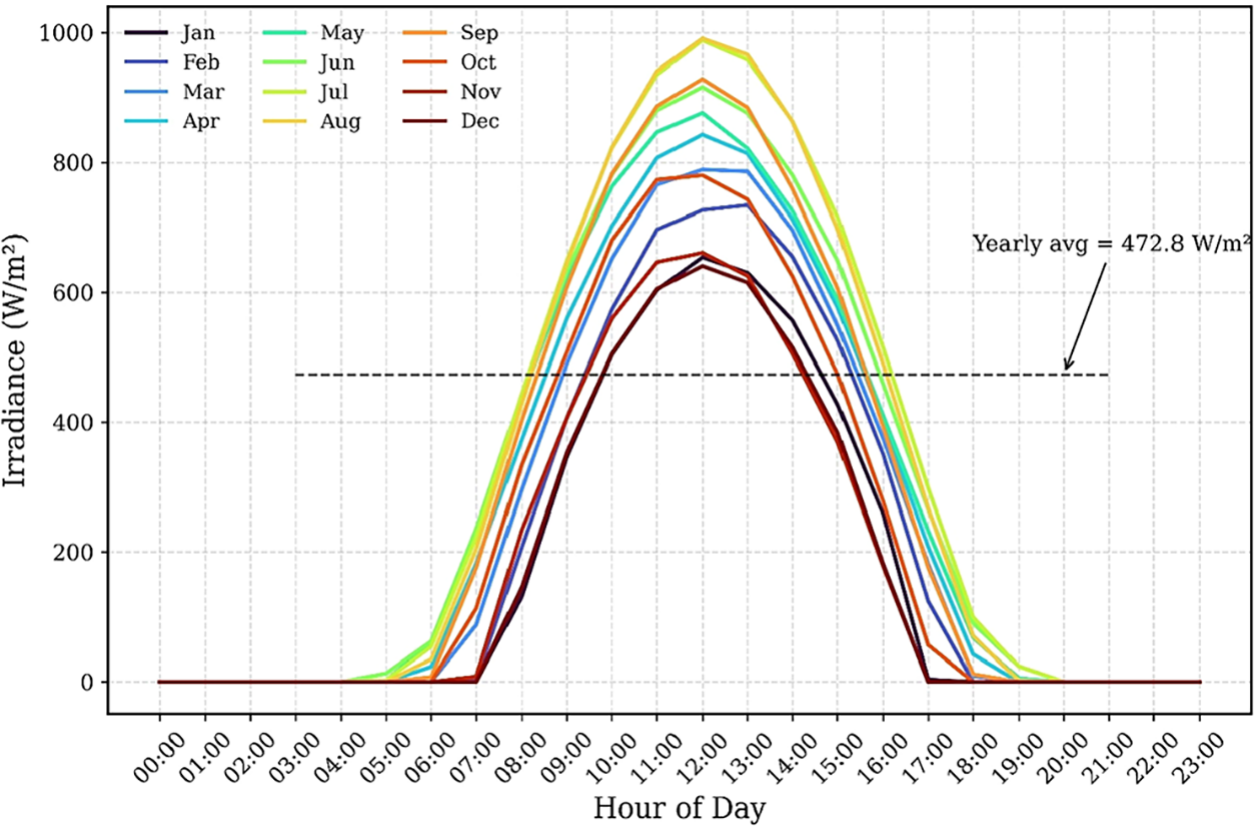
Daily solar GHI profile clustered by month and the yearly GHI average (472.8 W/m^2^). Data was sourced from the Photovoltaic Geographical Information System for Madrid, Spain (latitude 40.261°, longitude −3.803°) with a 35° plane tilt, and was utilized to size the integrated solar PV farm.

### 2.5 Electrolysis and Methanation

In Cases III and IV, the system is extended to include water electrolysis and CO_2_ methanation. Unlike the core chemical plant simulated in Aspen Plus, these units are formulated as equation-oriented modules implemented in Lua within the OSMOSE platform. The alkaline electrolyzer utilizes flexible electricity (during periods of low grid prices or high solar PV generation) to produce green hydrogen. This hydrogen is subsequently combined with the CO_2_ captured from the syngas conditioning step to produce synthetic natural gas CH_4_. Alternative CO_2_ utilization pathways exist, such as the CAMERE process, which employs the reverse water–gas shift (RWGS) reaction to convert CO_2_ and H_2_ into CO for additional methanol synthesis.^61^ However, in the present study, auxiliary methanation was selected due to thermodynamic and operational advantages within a flexible, renewable-powered framework. The RWGS reaction is highly endothermic and requires a continuous supply of high-temperature heat. In contrast, CO_2_ methanation is strongly exothermic, allowing the released heat to be effectively integrated into the plant’s steam network. Furthermore, because the electrolyzer is designed to operate flexibly and intermittently, feeding fluctuating amounts of RWGS-derived CO into the main methanol synthesis loop would disturb the required SN and compromise reactor stability. By operating an independent methanation module within OSMOSE, the flexible power-to-gas subsystem can be decoupled from the steady-state baseload methanol plant, while coproducing CH_4_ as a secondary energy vector.

### 2.6 Optimization Framework and Problem Definition

This section presents the plantwide energy integration approach^62^ to determine the MER in each configuration. The optimization framework adopted in this study is adapted from the methodology proposed by ref,^63^ which integrates sequential steps of process modeling, simulation, and optimization. This approach has been applied to the modeling and optimization of the biomass-to-methanol synthesis pathway integrated with CO_2_ capture. As discussed so far, the biomass-to-methanol synthesis system exhibits a complex and highly integrated configuration, where process streams are interconnected through recycle loops and extensive heat-recovery networks. Consequently, the process synthesis and optimization require a systematic and computationally efficient framework capable of identifying optimal process configurations within a reasonable time frame. In this context, the selection among the proposed utility units and process alternatives is formulated as an MILP problem. This formulation enables the identification of the most suitable configuration that minimizes both the operating costs and MER.

To calculate the MER for the methanol synthesis, the contribution of every hot and cold streams to overall heat balance must be represented on the respective composite curves.^64^ These curves are shifted by a minimum temperature difference contribution, which depends on the physical characteristics and heat transfer properties of the involved streams, to ensure feasible and realistic heat exchange. The subsequent energy integration and MER evaluation are conducted using the OSMOSE Lua platform developed by the Industrial Process and Energy Systems Engineering (IPESE) group at École Polytechnique Fédérale de Lausanne (EPFL).^65^ Given the possibility of both importing electricity from the grid and generating it internally (e.g., through the combustion of the purge stream or via the solar power plant), the system must balance external energy purchases against internal generation. Therefore, the optimization aims to minimize the overall utility cost by identifying the most economical pathway for minimum grid import. It must also be noted that outcomes entirely depend on the initial number of energy technologies and resources adopted, and the window of the appropriate operating conditions assumed (e.g., steam pressure levels, PV farm operation).

In the first stage, a comprehensive list of all feasible utility systems, such as the steam network, furnace, refrigeration unit, heat pump, and cogeneration system, is identified based on the analysis of the grand composite curve (GCC).^59^ These utility options are evaluated for their suitability to provide heating and cooling services across the process temperature ranges. The computational framework subsequently interfaces with Aspen Plus to exchange process data and formulate the MILP problem described in eqs 13–18. This optimization problem seeks to minimize the overall consumption of resources such as water, biomass, and electricity, and reduce the total operating cost of the methanol synthesis process, while satisfying the thermodynamic constraints imposed by the MER problem.^64^

AMPLⓇ CPLEX solver is utilized to solve this MILP problem. In this formulation, the integer variables, *y*_ω_, represent the existence or absence of any utility unit ω, while corresponding continuous load factors *f*_ω_ denote their operating levels. The optimization aims to determine these variables by minimizing the objective function given by the eq 13.

13 $$\underset{f_{\omega }\;,y_{\omega }\;R_{r}\;,W}{\min_{︸}}\left[{\left(f_{\omega }\cdot ṁ\cdot c\right)}_{\mathrm{woody}\;\mathrm{biomass}}+{\left(f_{\omega }\cdot V\cdot c\right)}_{\mathrm{water}}\pm {\left(f_{\omega }\cdot W\cdot c\right)}_{\mathrm{Elec}\;\mathrm{Grid}}-{\left(f_{\omega }\cdot W\cdot c\right)}_{\mathrm{Elec}\;\mathrm{Purge}}-{\left(f_{\omega }\cdot W\cdot c\right)}_{\mathrm{Elec}\;\mathrm{Solar}}-{\left(f_{\omega }ṁ\cdot c\right)}_{\mathrm{MeOH}}-{\left(f_{\omega }\cdot W\cdot c\right)}_{{\mathrm{CH}}_{4}\;\mathrm{marketed}}\right]\cdot t_{\mathrm{OP}}$$

subject to the constraints:

The heat balance at the temperature interval *r*

14 $$\sum_{\omega =1}^{N_{\omega }}f_{\omega }q_{\omega ,r}+\sum_{i=1}^{N}Q_{i,r}+R_{r+1}-R_{r}=0,\forall_{r}=1...N$$

Power balance for any production and consumption

15 $$\sum_{\omega =1}^{N_{\omega }}f_{\omega }W_{\omega }+\sum_{\mathrm{chemical}\;\mathrm{units}}W_{\mathrm{net}}+W_{\mathrm{imp}}-W_{\exp}=0$$

For any utility unit existence and load

16 $$f_{\min,\omega }y_{\omega }\leq f_{\omega }\leq f_{\max,\omega }y_{\omega },\forall_{\omega }=1...N_{\omega }$$

17 $$\sum_{\omega =1}^{N_{\omega }}y_{\omega }\leq 1,\mathrm{where}{\;y}_{\omega }\epsilon \left\{0,1\right\},\mathrm{and}{\;f}_{\omega }\geq 0,\forall_{\omega }=1...N_{\omega }$$

Feasible solution for MER

18 $$R_{1}=0,R_{N+1}=0,,R_{r}\geq 0\;\mathrm{and}\;W_{\mathrm{imp}}\leq 0,W_{\exp}\geq 0$$

where *N* represents the number of temperature intervals, determined by the supply and target temperatures of the process streams. *Q* denotes the heat exchanged between the process streams, where *Qi*,*r >* 0 corresponds to a hot stream releasing heat and *Qi*,*r <* 0 to a cold stream absorbing heat. *R* refers to the heat flow rate (kW) that is cascaded from higher (*r +* 1) to lower (*r*) intervals of temperature. The parameter *N*ω indicates the total number of utility units considered in the system, while *ṁ* represents the mass flow rate (kg h^–1^). In addition, *q* are the cooling or heating flow rates supplied by the utility systems (kW), and *W* is the produced power by the utility systems, chemical processes or exchanged with the grid (kW). *c* is the price of feedstock (€/kg), electricity consumed (€/kWh), or revenues from marketable products and byproducts. *t*_OP_ is the operation time (h).

The biomass-to-methanol is modeled in Aspen Plus, while the auxiliary subsystems, including the electrolyzer, carbon storage, methanation, and other utilities shown in Figure 2, are formulated as equation-oriented modules using Lua within the OSMOSE platform.^63^ Each module embeds the corresponding mass and energy balance relations following the multilayer integration approach adopted in OSMOSE,^63,66^ as illustrated in Figure 4. In this framework, process units and utility systems interact simultaneously through dedicated layers representing heat, steam, electricity, and material flow. The framework enables consistent evaluation of energy and resource exchanges across the system. For example, the electricity balance in the power layer ensures that the demand of process and auxiliary units is satisfied through either on-site generation (e.g., PV) or grid exchange when required.

**Figure 4:**
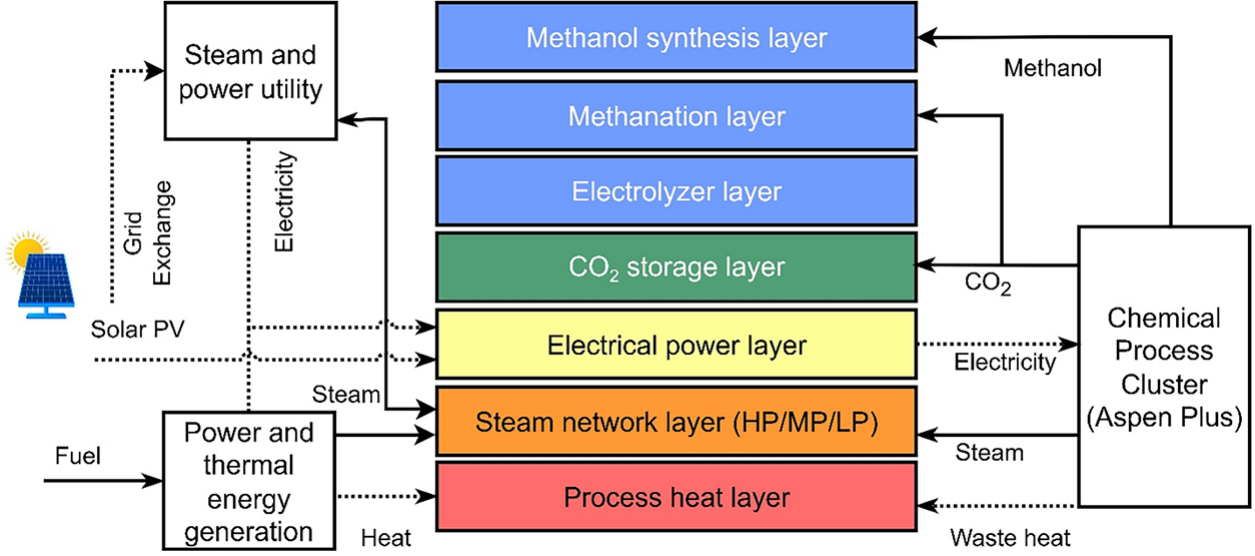
Multilayer integration framework implemented in the OSMOSE platform for the biomass-to-methanol system. The chemical process cluster is simulated in Aspen Plus, while the supporting subsystems, including electrolysis, methanation, CO_2_ storage, and energy utilities, are modeled as equation-oriented modules within OSMOSE. The framework links the electricity, steam, and heat layers with the process model to evaluate the simultaneous interaction of mass, energy, and utility flows across the system.

### 2.7 Economic Analysis

The economic performance of the integrated biomass-to-methanol system was evaluated through the estimation of both capital expenditures (CAPEX) and operating expenditures (OPEX). Equipment purchase costs for the main process units, biomass gasifier, air separation unit, water–gas shift reactor, CO_2_ capture unit, methanol synthesis loop, electrolyzer, and utility systems, were estimated by applying capacity scaling laws to reference costs reported in the literature.^10^ The scaling relation is given by eq 19.

19 $${\mathrm{cost}}_{\mathrm{new}}={\mathrm{cost}}_{\mathrm{base}}{\left(\frac{{\mathrm{capacity}}_{\mathrm{new}}}{{\mathrm{capacity}}_{\mathrm{base}}}\right)}^{R}$$

where Cost_new_ is the updated cost of any process equipment with the Capacity_new_ based on the specific volume/capacity of that equipment. The Cost_new_ is corrected based on the previously calculated cost Cost_base_ and the Capacity_base_ of the same processing equipment. Meanwhile, *R* is the power scaling factor, varying between 0.5 and 0.9, based on the type of process considered. For cash flow calculations, a plant lifespan of 20 years is assumed. Moreover, the total capital expenditure is divided between the first (60%) and second (40%) years. A decommissioning cost of 6% of the overall CAPEX is assumed. The unit investments depend on the size of the components (which follow from the Aspen modeling), by scaling from known scales in the literature, see Table S9. Meanwhile, costs associated with auxiliary equipment, including pumps, vessels, and compressors, together with operating expenditures (OPEX), are estimated according to the methodology proposed by ref.^67^ Comparative economic analyses of four cases were conducted to evaluate trade-offs among investment costs, operating expenditures, and product revenues, with the aim of identifying the configuration that yields the lowest MSP of methanol. The MSP is defined as the break-even selling price of methanol required to achieve a net present value of zero over the plant lifetime. This calculation accounts for annualized capital recovery, operating and maintenance expenditures, revenues from byproducts (e.g., CH_4_ sales or CO_2_ credits), and the specified discount rate. Under this definition, the MSP is conceptually equivalent to the levelized cost of methanol, representing the constant product price required to cover the total life-cycle cost of the process.

## 3 Results and Discussion

This section presents and discusses the simulation and optimization results of the integrated biomass-to-methanol system for the four process configurations defined in Section 2 (Cases I–IV). The analysis progressively builds from a reference electrified biomass-to-methanol pathway toward configurations incorporating renewable electricity integration and auxiliary carbon utilization, with results evaluated from both technical and energy-performance perspectives. Energy integration and economic implications are discussed subsequently.

### 3.1 Case I: Baseline Process Performance

Case I serves as the reference configuration, representing an electrified biomass-to-methanol process without solar electricity integration or auxiliary methanation. The overall mass balances for the lignocellulosic route and agri-food route are summarized in Tables 4 and 5, respectively, detailing the flow rates, operating conditions, and major gas compositions throughout each key processing step. Detailed component-level mass balance tables for all evaluated cases are additionally provided in the Supporting Information (Tables S3–S8). The feedstock enters the bubbling fluidized-bed gasifier at a rate of 16,666 kg h^–1^, accompanied by 15,000 kg h^–1^ of steam as the gasifying agent, the same for both feedstocks. The initial product streams from both gasification cases exhibit limited hydrogen content, with relatively low H_2_/CO ratios and SN, insufficient for direct methanol synthesis. Subsequent catalytic tar reforming effectively decomposes higher hydrocarbons and aromatics, enhancing the hydrogen concentration and improving the overall gas quality. Following reforming, both the H_2_/CO ratio and the SN increase significantly, indicating partial adjustment toward the range required for methanol synthesis. The HT-WGS reactor promotes the conversion of CO and H_2_O into additional H_2_ and CO_2_.

**Table 4:** Performance and Mass Balance of the Methanol Synthesis in Cases I and II Using Lignocellulosic Biomass Gasification (without Auxiliary Methanation)

stage	gasification		reforming	HT-WGS	lean syngas	pure CO_2_	MeOH synthesis		
	In	Out	Out	Out	Out	Out	MeOH	Water	Purge
Biomass (kg h^–1^)	16,666	-	-	-	-	-	-	-	-
water (kg h^–1^)	15,000	-	-	1873	-	-	-	-	-
T (C)	800	800	800	362	30	30	30	30	50
P (bar)	1	1	1	30	30	1	1	1	2
Total flow rate (kg h^–1^)	31,666	29,970	29,970	23,398	14,915	7873	13,723	70	1122
H_2_/CO	-	1.663	1.594	2.1	2.126	-	-	-	-
SN	-	0.466	1.169	1.17	2.04	-	-	-	-
Wet Mol Fraction									
H_2_	-	0.145	0.422	0.5722	0.657	0.030	4.5 × 10^–07^	0	0.491
CO	-	0.087	0.265	0.2724	0.309	0.040	7.2 × 10^–08^	-	0.025
CO_2_	-	0.071	0.051	0.1168	0.008	0.920	2.5 × 10^–03^	-	0.069
CH_4_	-	0.030	0.0179	0.022	0.024	-	9.2 × 10^–05^	-	0.361
H_2_O	-	0.608	0.240	0.0135	-	0.004	2.5 × 10^–03^	0.95	4.8 × 10^–05^
N_2_	-	9.0 × 10^–04^	2 × 10^–05^	2.5 × 10^–05^	-	-	-	-	-
C_2_H_4_	-	0.019	4.6 × 10^–06^	5.7 × 10^–06^	2.4 × 10^–04^	-	3.6 × 10^–05^	-	3.5 × 10^–03^
C_6_H_6_	-	2.0 × 10^–05^	1.4 × 10^–03^	1.7 × 10^–03^	-	-	-	-	-
C_10_H_8_	-	6.4 × 10^–03^	6.9 × 10^–04^	8.5 × 10^–04^	-	-	-	-	-
Char (gm/kg-biomass)	-	75.31	-	-	-	-	-	-	-
Ash (gm/kg-biomass)	-	25.36	-	-	-	-	-	-	-
Selexol	-	-	-	-	4.0 × 10^–08^	6.4 × 10^‑6^	-	-	-
MeOH	-	-	-	-	-	-	0.995	0.049	0.034

**Table 5:** Performance and Mass Balance of the Methanol Synthesis in Cases I and II Using Agri-Food Waste Gasification (without Auxiliary Methanation)

stage	gasification		reforming	HT-WGS	Lean syngas	Pure CO_2_	MeOH synthesis		
	In	Out	Out	Out	Out	Out	MeOH	Water	Purge
Biomass (kg h^–1^)	16,666	-	-	-	-	-	-	-	-
water (kg h^–1^)	15,000	-	-		-	-	-	-	-
T (C)	800	800	800	354	30	30	30	30	50
P (bar)	1	1	1	30	30	1	1	1	2
Total flow rate (kg h^–1^)	31,666	28,116	28,116	21,029	14,426	6080	13,313	67	1044
H_2_/CO	-	1.908	1.636	2.050	2.07	-	-	-	-
SN	-	0.736	1.306	1.306	2.04	-	-	-	-
Wet Mol Fraction									
H_2_	-	0.147	0.429	0.586	0.656	0.046	5.7 × 10^–07^	-	0.454
CO	-	0.077	0.262	0.286	0.316	0.06	1.3 × 10^–07^	-	0.037
CO_2_	-	0.052	0.037	0.092	0.003	0.863	1.1 × 10^–03^	-	0.033
CH_4_	-	0.021	0.012	0.015	0.016	8.7 × 10^–03^	8.7 × 10^–05^	-	0.287
H_2_O	-	0.635	0.250	9.5 × 10^–03^	-	3.1 × 10^–03^	4.7 × 10^–04^	0.947	1.4 × 10^–05^
N_2_	-	7.6 × 10^–03^	5.4 × 10^–03^	6.9 × 10^–03^	9.5 × 10^–04^	7.6 × 10^–03^	-	-	0.132
C_2_H_4_	-	0.0203	1.4 × 10^–03^	1.8 × 10^–03^	1.5 × 10^–04^	1.6 × 10^–02^	2.7 × 10^–05^	-	2.5 × 10^–03^
C_6_H_6_	-	3.0 × 10^–02^	2.1 × 10^–04^	2.7 × 10^–04^	-	1.4 × 10^–04^	-	-	-
C_10_H_8_	-	6.6 × 10^–03^	4.8 × 10^–06^	6.0 × 10^–06^	-	-	-	-	-
Char (gm/kg-biomass)	-	81.554	-	-	-	-	-	-	-
Ash (gm/kg-biomass)	-	2.498	-	-	-	-	-	-	-
Selexol	-	-	-	-	3.9 × 10^–08^	6.7 × 10^–06^	-	-	-
MeOH	-	-	-	-	-	-	0.998	0.052	0.051

Following the removal of CO_2_ in the Selexol unit, a purified syngas suitable for methanol synthesis is obtained. For the lignocellulosic route, the conditioned syngas reaches an H_2_/CO ratio of approximately 2.13 and an SN of 2.04, while corresponding values of 2.07 and 2.05 are obtained for the agri-food route. These values fall well within the optimal range for the methanol synthesis. Minor differences between feedstocks originate from their intrinsic elemental composition, with the agri-food feedstock exhibiting a slightly higher oxygen content and CO_2_ formation during gasification. However, after reforming and shifting reactions, the resulting syngas compositions converge to nearly identical specifications.

The conditioned syngas is subsequently converted in the methanol synthesis loop to a liquid product containing 99.53 wt % methanol for the lignocellulosic route and 99.83 wt % methanol for the agri-food route, with minor amounts of water and light gases produced as byproducts. The methanolization reactor effluent is separated into liquid and gaseous streams, where the purge streams primarily consist of unreacted H_2_, CH_4_, and CO_2_, preventing the accumulation of inert and light hydrocarbons within the loop. The overall carbon oxides conversion (CO and CO_2_ into methanol) obtained is 96.9 and 97% for lignocellulosic biomass gasification route and agri-food gasification route, respectively, which is consistent with the 96% overall conversion reported by ref^68^ for fixed bed based isothermal methanol synthesis loop.

The mass balance for Cases I and II in the agri-food gasification route is given in Table 5.

For stoichiometric consistency, H_2_ demand for complete CO_*x*_ conversion is evaluated by eq 20

20 $$H_{2}^{\mathrm{req}}=2n_{\mathrm{CO}}+3n_{{\mathrm{CO}}_{2}}$$

where *n*_CO_ and *n*_CO_2__ are molar flow rates for CO and CO_2_, respectively. Based on this, the hydrogen demand (H_2_^req^) was calculated as 890.74 kmol/h for the route based on lignocellulosic feedstock and 856.84 kmol/h for that based on agri-food waste. The syngas feed to the methanol synthesis loop was adjusted to include a slight excess of hydrogen relative to stoichiometric requirements in order to mitigate equilibrium limitations. As a result, hydrogen flow rates of approximately 909 kmol h^–1^ for lignocellulosic feedstock and 876 kmol h^–1^ for agri-food feedstock were supplied to the synthesis loop. Consequently, the overall H_2_-to-methanol conversion reached 97.91% for the lignocellulosic route and 96.8% for the agri-food route, which are consistent with the ∼98% reported by.^68^ These values remain unchanged across all four cases (Case I to Case IV), as the methanol synthesis section is identical in each case.

The carbon conversion efficiency of the process was also evaluated using eq 21.

21 $$\eta_{C}=\left(\frac{\sum_{i}C_{\mathrm{in}\;\mathrm{product}}}{C_{\mathrm{in}\;\mathrm{feed}}}\right)\times 100$$

where *C*_in feed_ is the carbon in the dry biomass and *C*_in product_ is the carbon incorporated into methanol (and other byproducts, if any). The results yield carbon efficiencies of 61.6% for the lignocellulosic route and 52.63% for the agri-food route, which are substantially higher than the 33.3% reported by Harris et al. for biomass gasification-based methanol synthesis.^69^ The lower efficiencies reported in biomass gasification-to-methanol systems described in the literature primarily arise from the diversion of syngas for tar reforming and process heat generation, leading to carbon losses through flue gas emissions. In contrast, the present study employs Joule (resistive) heating to supply thermal energy to both the gasifier and tar reformer, thus eliminating the need for syngas combustion and preserving a greater fraction of infeed carbon to methanol. Although electrolysis-based pathways, such as indirect or direct CO_2_-to-methanol conversion, can achieve carbon efficiencies exceeding 91–99%, the 61.6% efficiency attained in this work represents a significant improvement over previously reported combustion-heated biomass gasification routes.^69^ This underscores the effectiveness of electrified heat integration in enhancing carbon utilization and improving the overall performance of biomass-derived methanol synthesis.

The electrical demand in Case I amounts to 9.66 MW for the lignocellulosic route and 13.75 MW for the agri-food route. In the lignocellulosic configuration, purge gas is recovered for internal power generation, whereas in the agri-food route, it is utilized for feedstock drying. Case I therefore establishes the reference electrified biomass-to-methanol configuration against which the effects of renewable electricity integration and auxiliary methanation are assessed in the following sections.

### 3.2 Case II: Integration of Solar Electricity

Case II builds directly upon the reference configuration described in Case I and is thermochemically identical in terms of process layout, material flows, and conversion performance. The sole distinction lies in the electricity supply strategy, where a fraction of the plant’s electrical demand is met by a PV solar farm in combination with grid electricity, instead of relying exclusively on grid power. The integration of solar electricity does not alter the syngas composition, hydrogen demand, methanol synthesis conditions, or carbon conversion efficiency, which therefore remain unchanged relative to Case I. Figures S1 and S2 illustrate the average hourly electricity balance throughout the year for the lignocellulosic and agri-food waste routes, respectively, showing the contribution of PV solar generation and grid electricity in meeting the plant’s power demand. The profiles represent monthly average diurnal variations, highlighting periods of grid import and electricity export resulting from surplus solar generation. While process performance indicators are identical to those of the reference case, the inclusion of PV power affects the economic structure of the system through changes in capital investment and operating costs associated with electricity supply. These differences are quantified and discussed in detail in the economic analysis section.

### 3.3 Case III: Operation with Auxiliary Methanation

Case III extends the reference configuration by integrating an alkaline electrolyzer and an auxiliary methanation unit, enabling the conversion of captured CO_2_ into synthetic methane using electrolytic hydrogen. Unlike Cases I and II, the operation of these additional units is highly sensitive to electricity price variability. Consequently, a time-resolved optimization framework was adopted to evaluate flexible operation under fluctuating electricity market conditions. Tables 6 and 7 present the monthly optimization results for Case III, corresponding to lignocellulosic and agri-food feedstocks, respectively. In this scenario, the OSMOSE platform performs a year-round optimization using monthly electricity price profiles representative of the Spanish power market, characterized by lower prices during spring and summer (March–October) and higher prices during autumn and winter (November–February). The optimization strategy exploits this variability by operating the electrolyzer primarily during low-price periods to produce green hydrogen, while captured CO_2_ is compressed and liquefied for storage during high-price months. The stored CO_2_ is subsequently combined with electrolytic hydrogen in the methanation unit to produce value-added methane.

**Table 6:** Process Performance and Mass Balance of the Lignocellulosic Biomass-to-Methanol Route with Methanation and Electrolysis in Case III

months	march–october	november–february
biomass (kg h^–1^)	16,666	16,666
MeOH (kg h^–1^)	13,723	13,723
Electricity (kW)		
Cooling tower	851	475
Electrolyzer	117,291	0
CO_2_ storage	0	787
CO_2_ Compression	119	0
Methanator CO_2_ comp	621	0
MeOH synthesis	12,719	12,719
BFBG	4333	4333
Reformer	4311	4311
Steam Turbine (kW)	8266	4579
Water (m^3^/h)		
Cooling tower	53.324	29.773
Electrolyzer	19.193	0
MeOH synthesis	10.637	10.637
Methanation		
H_2_ (kg h^–1^)	2132	0
Oxygen (kg h^–1^)	16833	0
CO_2_ storage (kg h^–1^)	0	7873
CO_2_ compression (kg h^–1^)	3856	0
Methanator CH_4_ (kW)	59,238	0

**Table 7:** Process Performance and Mass Balance of the Agri-Food-to-methanol Configuration with Auxiliary Methanation and Electrolysis in Case III

	march–october	november–february
biomass (kg h^–1^)	16,666	16,666
MeOH (kg h^–1^)	13,313	13,313
Electricity (kW)		
Cooling tower	722	432
Electrolyzer	90,589	0
CO_2_ storage	0	608
CO_2_ Compression	92.35	0
Methanator CO_2_ comp	480.32	0
MeOH synthesis	14,569	14,569
BFBG	4333	4333
Reformer	4311	4311
Steam turbine (kW)	7835	3985
Water (m^3^/h)		
Cooling tower	45.217	27.051
Electrolyzer	14.823	0
MeOH synthesis	5.589	5.589
Methanation		
H_2_ (kg h^–1^)	1647	0
Oxygen (kg h^–1^)	13,028	0
CO_2_ storage (kg h^–1^)	0	6080
CO_2_ compression (kg h^–1^)	2978	0
Methanator CH_4_ (kW)	45,752	0

A comparison between Tables 6 and 7 indicates that the lignocellulosic configuration achieves slightly higher methanol and methane production than the agri-food route under identical biomass feed rates. Methanol production reaches 13,723 kg h^–1^ for lignocellulosic biomass and 13,333 kg h^–1^ for agri-food waste, corresponding to a difference of approximately 3%. This difference reflects variations in feedstock composition and resulting syngas carbon availability.

The electrical demand in Case III is primarily associated with the electrolyzer, methanol synthesis loop, CO_2_ compression and liquefaction units, cooling systems, and auxiliary power requirements, while electrically supplied process heat is used exclusively for the BFB gasifier and the reformer. For Case III, the overall electrical consumption pattern is similar for both feedstocks when accounting for seasonal operation. In this configuration, the integrated steam turbine network recovers process heat and generates between 4.57 and 8.26 MW for the lignocellulosic route and 3.98 to 7.83 MW for the agri-food route, with higher power generation occurring during summer operation. This internal power generation partially offsets net imported electricity. During months with low electricity prices, when the electrolyzer is actively operated, total electrical consumption increases markedly, reaching 117.3 MW for the lignocellulosic biomass gasification route and 90.6 MW for the agri-food gasification route. In contrast, during high-price periods when the electrolyzer is idle, the CO_2_ compression and liquefaction units remain operational to store captured CO_2_, which is subsequently utilized during the summer operation in the methanation process. On average, the electrolyzer consumed 19.13 m^3^h^–1^ of water for the lignocellulosic biomass gasification route and 14.82 m^3^h^–1^ for the agri-food route, generating approximately 2.13 tonne h^–1^ (lignocellulose route) and 1.65 tonne h^–1^ (agri-food route) of H_2_. This H_2_ was combined with CO_2_ from capture and storage to produce CH_4_ in the methanator, contributing a secondary energy vector that enhances the overall carbon valorization and plant revenue.

To quantify the contribution of auxiliary methanation, methane production is evaluated on both mass- and energy-referenced bases. The methane mass yield is defined relative to the biomass feed rate, while the methane energy efficiency accounts for both biomass chemical energy and net electricity input by eq 22

22 $$Y_{CH_{4},\mathrm{mass}}=\frac{{ṁ}_{CH_{4}}}{{ṁ}_{\mathrm{feed}\;\mathrm{stock}}}\times 100\;\mathrm{and}\;\eta_{CH_{4},\mathrm{energy}}=\frac{{Ė}_{CH_{4}}}{{Ė}_{\mathrm{total}}}\times 100$$

The resulting methane yields are listed in Table 8. The lignocellulosic route exhibits a higher methane mass yield (25.6%) than the agri-food route (19.8%), reflecting its higher carbon conversion efficiency. When methanol and methane products are considered together, the overall carbon utilization reaches 82.9% for lignocellulosic biomass and 68.4% for agri-food waste.

**Table 8:** Mass and Energy Yield of the CH_4_ Based on the Input Feedstock for Case III

feedstock	CH_4_ (kW)	CH_4_ (kg h^–1^)	mass yield (%)	energy yield (%)
Lignocellulosic residue	59,238	4265	17.2	22.4
Agri-food waste	45,752	3294	13.3	16.3

### 3.4 Case IV: Combined Solar and Methanation Integration

Case IV combines the features of Cases II and III by integrating auxiliary methanation with on-site solar PV electricity generation. This configuration maintains the same feedstock flow rate of 16,666 kg h^–1^ for both lignocellulosic biomass and agri-food gasification routes, as given in the Table 9. Compared to Case III, the integration of solar PV reduces exposure to seasonal electricity price variability by supplying a significant fraction of the plant’s electricity demand with low-cost renewable power. As a result, the operation of the electrolyzer and methanation units becomes less constrained by market price fluctuations. The inclusion of the PV significantly alters the internal power balance. The PV modules generate 93.6 MW (lignocellulosic route) and 91.0 MW (agri-food route) of electricity, supplying most of the process demand during the day. The steam turbine continues to recover process heat, generating 7.9 MW (lignocellulose route) and 7.7 MW (agri-food route). In Case IV, the methanator output is 39.8 MW of CH_4_ for the lignocellulosic route and 37.2 MW for the agri-food route, with a steady CO_2_ conversion.

**Table 9:** Process Performance of Lignocellulosic Biomass and Agri-Food Gasification Routes in Case IV

parameter	lignocellulosic	agri-food
Input rate (kg h^–1^)	16,666	16,666
MeOH (kg h^–1^)	13,723	13,313
Electricity (kW)		
Cooling tower	727	667
Electrolyzer	78,730	73,711
Methanator CO_2_ compression	622	40
MeOH synthesis	12,719	14,569
Gasification joule heating	4333	4333
Tar reforming joule	4311	4311
Electricity PV module	93,574	91,042
Steam turbine	7912	7671
Water (m^3^/h)		
Cooling tower	29.77	41.82
Electrolyzer	12.88	12.06
Methanol synthesis	10.64	5.59
Methanation (CH_4_) and Electrolyzer (O_2_) exports		
H_2_ consumption (to methanator) (kg h^–1^)	1431	1340
Oxygen exported (from electrolyzer) (kg h^–1^)	11,298	10,601
CO_2_ compression (to methanator) (kg h^–1^)	7873	6080
Methanator CH_4_ production (kW)	39,762	37,228

Table 10 summarizes the CH_4_ mass and energy yield metrics for Case IV as per eq (24), for both lignocellulosic and agri-food feedstocks. For both feedstocks, CH_4_ is produced via the methanation of captured CO_2_ using electrolytic hydrogen, with the resulting CH_4_ power output of 39.8 MW for lignocellulosic biomass and 37.2 MW for agri-food waste. These values correspond to CH_4_ mass flow rates of 2863 and 2680 kg h^–1^, respectively. The methane mass yield is expressed relative to the biomass feed rate and indicates the amount of CH_4_ produced per unit mass of biomass processed. Under identical biomass feed rates, lignocellulosic residues achieve a slightly higher mass yield (17.2%) than agri-food waste (16.2%), reflecting differences in feedstock composition and carbon availability. The CH_4_ energy yield accounts for both the chemical energy of the biomass and the net electrical energy input required for electrolytic hydrogen production and process operation. When defined on this basis, the lignocellulosic route attains a higher overall CH_4_ energy yield (23.6%) compared to the agri-food route (18.7%), indicating more favorable energy conversion performance under the solar-assisted configuration of Case IV.

**Table 10:** Mass and Energy Yield of the CH_4_ Based on the Input Feedstock for Case IV

feedstock	CH_4_ (kW)	CH_4_ (kg h^–1^)	mass yield (%)	energy yield (%)
Lignocellulosic residue	39,762	2863	17.2	23.6
Agri-food waste	37,228	2680	16.2	18.7

### 3.5 Energy Integration Analysis

Figures 5 and 6 present the energy integration results for the methanol synthesis configuration of the gasification-based routes for lignocellulosic and agri-food residues, respectively. The composite curves shown in Figures 5(a) and 6(a) correspond to the process configuration including auxiliary methanation (Case III), which is also thermally equivalent to Case IV, as the difference between these two cases lies solely in the source of electricity supply. Since Cases I and II share the same process layout without methanation, and Cases III and IV share an identical process configuration with methanation, differing only in the electricity sourcing strategy (grid-based or solar-assisted), the underlying heat-integration characteristics remain unchanged across all scenarios. Energy integration is governed by stream temperature levels, phase changes, and process heat duties, which are independent of the electricity supply source. Consequently, the composite curves presented in Figures 5 and 6 are representative of the overall methanol synthesis process for all four cases and adequately capture the maximum internal heat-recovery potential of the system.

**Figure 5:**
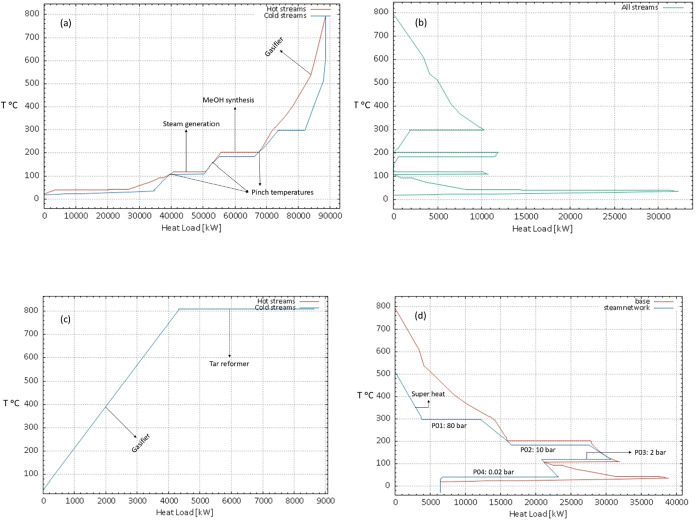
Composite curves of the biomass (lignocellulosic)-to-methanol integrated route: (a) integrated composite curves, (b) grand composite curve, (c) heat requirement for gasifier and tar reformer, and (d) turbine steam network.

**Figure 6:**
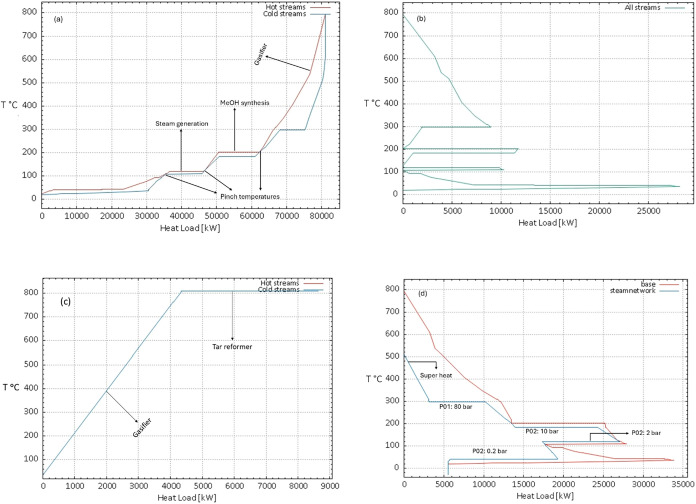
Composite curves of the biomass (agri-food)-to-methanol integrated route: (a) integrated composite curves, (b) grand composite curve, (c) heat requirement for gasifier and tar reformer, and (d) turbine steam network.

In both feedstock routes, the gasification section dominates the high-temperature region and constitutes the principal heat-demanding unit. Conversely, substantial recoverable heat is available from the methanol synthesis loop and the associated exothermic reaction stages, which can be effectively utilized for process heating and steam generation. The identified utility pinch temperatures, ranging approximately from 100 to 210 °C, define the boundary between heat-recovery zones and regions requiring external hot utilities.

Below the pinch, internal heat recovery is maximized through feed preheating and steam generation, whereas above the pinch, the process primarily relies on external heat supply. For the lignocellulosic route, the total recoverable heat load reaches approximately 88 MW under the reference operating conditions, while the agri-food residue route exhibits a slightly lower value of approximately 82 MW, mainly due to the reduced syngas flow rate and, consequently, lower overall thermal load entering the methanol synthesis loop. Despite these quantitative differences, the overall shapes and characteristics of the composite curves remain similar for both feedstocks.

Figures 5(b) and 6(b) present the grand composite curves (GCCs) for the lignocellulosic and agri-food gasification routes, respectively, providing a compact representation of the net heat surplus and deficit of the process across temperature intervals after maximum internal heat recovery. Horizontal plateaus in the GCC correspond to temperature ranges where heat is available or required at nearly constant temperature, typically associated with phase-change processes and steam generation or utilization at specific pressure levels. For the lignocellulosic gasification route, a net heat surplus is observed up to approximately 300 °C, indicating significant potential for medium-pressure steam generation and intermediate-temperature process heating. Above this temperature, the GCC reveals a net heat deficit, primarily associated with the gasification and reforming sections, which dominate the high-temperature energy demand and necessitate external heat supply. At lower temperatures (<100 °C), the GCC indicates the presence of low-grade excess heat, which can be exploited for feed preheating or ancillary thermal services. The GCC corresponding to the agri-food gasification route exhibits a very similar overall profile, confirming that both feedstock pathways can be effectively integrated using comparable steam networks and utility structures. However, the agri-food route shows a slightly reduced magnitude of excess heat, consistent with its lower syngas throughput and overall thermal load.

Figures 5(c) and 6(c) show the electrical heating demand associated with the gasifier and tar reformer, which are the main endothermic units. The heating profile increases from ambient to ∼800 °C, supplied by Joule elements. Both lignocellulosic and agri-food gasification routes (in all four cases) require 4333 and 4311 kW of electrical input to maintain the operating temperature of the gasifier and the tar reformer, respectively. Figures 5(d) and 6(d) demonstrate the integration of a multipressure steam network within the process for power generation. The implemented superstructure defines four pressure levels: P01 (80 bar), high-pressure steam generation and superheating; P02 (10 bar), medium-pressure steam; P03 (2 bar), low-pressure steam; and P04 (0.02 bar), turbine condenser, identified for optimal power generation. Each level is characterized by a superheating temperature difference, minimum temperature difference contribution, and steam turbine efficiency (if installed). The OSMOSE Lua platform connects these steam levels through turbine expansion and condensate return. Steam turbine electrical power is case-dependent because it reflects the electricity cogeneration schedule determined by the scenario and OSMOSE Lua optimization.

### 3.6 Economic Analysis

Tables 11 and 12 summarize the CAPEX normalized per unit dry biomass capacity (k€/t_DB_) for the four configurations (Cases I–IV) and two feedstocks at a 16,666 kg h^–1^ plant basis. For the lignocellulosic to methanol synthesis, Case I totals 250.36 k€/t_DB_, which is dominated by the methanol synthesis and purification, followed by the syngas conditioning by reforming and HT-WGSR. Adding solar energy in Case II increases CAPEX to 274.51 k€/t_DB_. Similarly, by adding methanation and CO_2_ storage in the Case III raises CAPEX to 348.52 k€/t_DB_ via the methanator with the highest block cost of 98.16 k€/t_DB_. Case IV brings the largest jump to 580.52 k€/t_DB_, driven by utility-scale PV together with the methanator. Similarly, from Table 12 CAPEX for the agri-food route, it can be seen that the investment is higher than the Case I, totaling 280.36 k€/t_DB_, because pretreatment is substantially larger than in the lignocellulosic route, reflecting rigorous drying requirements. Case II with PV rises by 9.1% to 305.79 k€/t_DB_. Case III with methanation increases to 376.47 k€/t_DB_. Similarly, Case IV, just like for the Lignocellulosic route, has the highest CAPEX. The highest capital contributions are for the photovoltaic plant for the electrical power because this includes the alkaline electrolyzer for H_2_ generation. The electrolyzer price 300 €/kW is selected based on the study as per Kourkoumpas et al.^70^ Across both feedstocks, CAPEX is anchored by the methanol synthesis loop, WGSR/reforming, and gasification sections, while PV integration and auxiliary methanation constitute the main contributors to incremental capital expenditure. But the agri-food gasification route has a higher pretreatment CAPEX, as shown in Table 11.

**Table 11:** CAPEX and OPEX for All Cases of Lignocellulose-to-Methanol Synthesis Route

	case I	case II	case III	case IV
Capital Cost (*10^3^ €/t_DB_)				
Pretreatment	14.418	14.418	14.418	14.418
Gasifier	49.748	49.748	49.748	49.748
Reforming and WGSR	58.479	58.479	58.479	58.479
CO_2_ Capturing	31.260	31.260	31.260	31.260
CO_2_ Compression	8.684	8.684	8.684	8.684
MeOH synthesis and Purification	67.321	67.321	67.321	67.321
Power generation	20.445	20.445	20.445	20.445
Solar Power	-	24.154	-	231.996
Methanation	-	-	98.161	96.181
Manufacturing Cost (€/t_DB_)				
Cost of Labor	36.236	36.296	36.355	36.415
Utility cost	41.064	1.863	400.650	183.188
Waste treatment cost	19.551	19.427	19.427	19.427
Feedstock cost	120	120	120	120
Land rent	-	0.213	-	2.137

**Table 12:** CAPEX and OPEX for All Cases of Agri-Food-to-Methanol Synthesis Route

	case I	case II	case III	case IV
CAPEX (*10^3^ €/t_DB_)				
Pretreatment	52.695	52.695	52.695	52.695
Gasifier	57.539	57.539	57.539	57.539
Reforming and WGSR	56.677	56.677	56.677	56.677
CO_2_ Capturing	26.520	26.520	26.520	26.520
CO_2_ Compression	8.596	8.596	8.596	8.596
MeOH synthesis and Purification	67.580	67.580	67.580	67.580
Power generation	10.750	10.750	10.750	10.750
Solar Power	-	25.433	-	227.605
Methanation	-	-	96.110	95.924
OPEX (€/t_DB_)				
Cost of Labor	36.236	36.296	36.296	36.415
Utility cost	57.456	29.424	328.133	165.152
Waste treatment cost	19.427	19.427	19.427	19.427
Feedstock cost	120	120	120	120
Land rent	-	0.214	-	2.167

The OPEX is expressed in €/t_DB_, representing the annual operating cost (€/year) normalized by the total annual biomass input (t_DB_/year). This allows direct comparison of the economic performance on a per-tonne-of-biomass basis. The OPEX (€/t_DB_) has been decomposed into labor, utilities, waste treatment, feedstock, and land rent (for PV cases). For both feedstocks, the Case I OPEX has feedstock cost and utilities cost as major components. Case II slashes the utility bill to 1.86 €/t_DB_ for the lignocellulose case, lowering total OPEX to 197.80 €/t_DB_, but for the agri-food, the utility cost has been reduced to 29.42 €/t_DB_ from the 57.46 €/t_DB_. This shows the higher utility requirements in the agri-food. In Case III, methanation is utility-intensive, lifting utilities to 400.65 and 328.13 €/t_DB_ for lignocellulosic and agri-food, respectively. Case IV reduces the utility-related costs of Case III to 183.19 €/t_DB_ and 165.15 €/t_DB_ for the lignocellulosic and agri-food routes, respectively; however, these values remain substantially higher than those of the first two cases. Land rent appears only with PV and remains less than 2.14 €/t_DB_. Overall, the results reveal that while core units drive the base capital cost, the choice of solar integration and methanation technologies shifts the balance between CAPEX and OPEX, strongly influencing the MSP of methanol.

Table 12 summarizes the CAPEX and OPEX breakdown for the agri-food residue-to-methanol route across all four cases. As expected, capital costs associated with the core process units, pretreatment, gasification, reforming/WGSR, CO_2_ capture, and methanol synthesis, remain identical across scenarios, while additional CAPEX is introduced only through solar power integration (Cases II and IV) and auxiliary methanation (Cases III and IV). Operating costs are primarily differentiated by utility consumption, which emerges as the dominant variable across cases. Case II exhibits the lowest utility cost (29.42 €/t_DB_), reflecting the replacement of grid electricity with solar power for the Case I process configuration. In contrast, Case III shows a pronounced increase in utility costs (328.13 €/t_DB_), driven by the substantial electricity demand of hydrogen production via electrolysis required for the methanation unit. Combining solar power integration with methanation in Case IV partially offsets this increase, reducing the utility cost to 165.15 €/t_DB_ through internal supply of low-cost electricity. However, utility costs in Case IV remain significantly higher than in the nonmethanation cases, indicating that the electricity demand associated with the electrolyzer–methanator system remains a major operating cost contributor even under solar-assisted operation.

Figures 7 and 8 present the results of the one-variable-at-a-time sensitivity analysis of the MSP of methanol for all investigated cases and both feedstocks. Each tornado diagram illustrates the variation in MSP induced by changes in key economic and technical parameters around the base value, with dashed vertical lines indicating the base MSP with and without CO_2_ credits.

**Figure 7:**
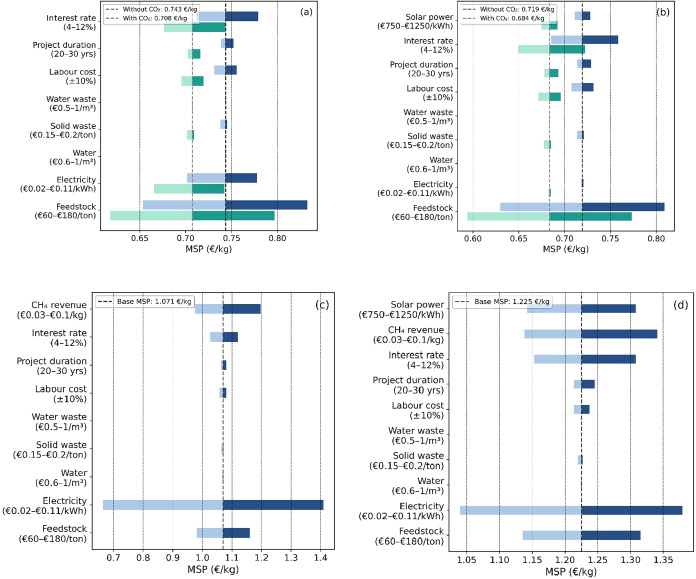
One-variable-at-a-time sensitivity analysis of the MSP of methanol for the lignocellulosic biomass-to-methanol route. Tornado diagrams illustrate the impact of variations in key economic and technical parameters on the MSP for four process configurations: (a) Case I, (b) Case II, (c) Case III, and (d) Case IV. Dashed vertical lines indicate the base MSP values with and without CO_2_ credit, while horizontal bars represent the change in MSP resulting from variations in the selected parameters around their base values.

**Figure 8:**
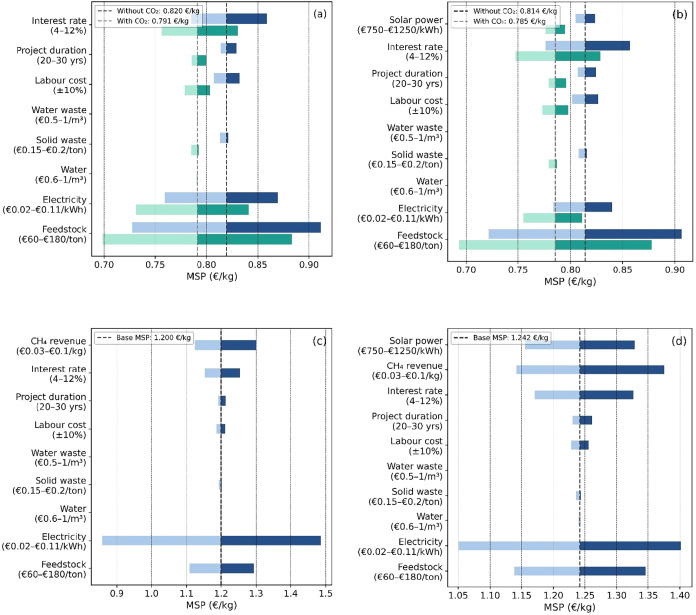
One-variable-at-a-time sensitivity analysis of the MSP of methanol for the agri-food residue-to-methanol route. Tornado diagrams illustrate the impact of variations in key economic and technical parameters on the MSP for four process configurations: (a) Case I, (b) Case II, (c) Case III, and (d) Case IV. Dashed vertical lines indicate the base MSP values with and without CO_2_ credit, while horizontal bars represent the change in MSP resulting from variations in the selected parameters around their base values.

Case I (Figures 7a and 8a) corresponds to the reference electrified biomass-to-methanol configuration. For the lignocellulosic route (Figure 7a), the base MSP is 0.743 €/kg without CO_2_ credit, which decreases to 0.708 €/kg when the CO_2_ credit is included. For the agri-food route (Figure 8a), the MSP is slightly higher, reflecting its greater pretreatment and capital requirements, resulting in a base MSP of 0.820 €/kg without CO_2_ credit, which decreases to 0.791 €/kg when the CO_2_ credit is included. In both feedstocks, the MSP remains substantially above the benchmark price of fossil-based methanol (≈0.30 €/kg).^71^ Across both feedstocks, the MSP in Case I is most sensitive to biomass feedstock price, followed by electricity price and interest rate. A reduction in feedstock cost to 60 €/t lowers the MSP to approximately 0.618 €/kg for lignocellulosic biomass and 0.70 €/kg for agri-food residues, indicating a similar relative response despite different baseline costs.

Case II (Figures 7b and 8b) evaluates partial substitution of grid electricity with solar power. For the lignocellulosic route, the MSP decreases to 0.719 €/kg without CO_2_ credit and 0.684 €/kg with CO_2_ credit, while for the agri-food route, the corresponding values are 0.814 €/kg and 0.785 €/kg. Compared to Case I, the sensitivity of the MSP to electricity price is markedly reduced, particularly for the lignocellulosic route, as the integration of PV power lowers dependence on grid electricity. Feedstock cost remains the dominant driver of MSP variability, with interest rate emerging as the next most influential parameter. Feedstock cost continues to exert the largest overall influence on the economics. In Case I, the electricity prices were the second most sensitive variable in the MSP of methanol synthesis.

The economic potential of the solar power plant integrated in Case II c, with an installed solar power capacity corresponding to a land use of 0.19 km^2^. The land requirements by solar PV farm was adopted by Menéndez et al.,^72^ whereas the solar power generation was calculated using daily average solar irradiation data by ref.^60^ At a grid electricity price of 0.07 €/kWh, this translates into annual savings of about €2.87 million, demonstrating the strong economic benefit of partial electrification via solar integration. Depending on the specific investment cost of the solar installation, the levelized cost of electricity ranges between 0.006 €/kWh for 750 €/kW and 0.011 €/kWh for 1250 €/kW, both of which are substantially below the prevailing grid price. Moreover, it shows that after incorporating the PV for the methanol synthesis, the minimum production cost can be reduced comparatively to Case I.

Case III (Figures 7c and 8c) introduces auxiliary methanation, increasing both capital investment and electricity demand due to electrolytic hydrogen production. As a result, the base MSP rises to 1.07 €/kg for lignocellulosic biomass and 1.20 €/kg for agri-food residues, significantly exceeding the values obtained in Cases I and II. In this configuration, electricity price becomes the most influential parameter, with MSP variations ranging from 0.66 to 1.41 €/kg for lignocellulosic biomass and 0.86 to 1.49 €/kg for agri-food residues. Feedstock price and CH_4_ revenue exert secondary but still significant effects, each inducing MSP variations of approximately ± 9%.

Case IV (Figures 7d and 8d) combines auxiliary methanation with large-scale PV integration and results in MSP values of 1.225 €/kg for the lignocellulosic route and 1.242 €/kg for the agri-food route. Among the investigated configurations, this case exhibits the highest MSP, primarily due to the substantial capital investment associated with the PV installation, as evidenced by the CAPEX breakdowns in Tables 11 and 12. Although solar power integration significantly reduces reliance on grid electricity, the overall MSP remains sensitive to electricity price because of the high electrical demand of the electrolyzer–methanator system and the additional capital burden introduced by the PV infrastructure. Consequently, while Case IV improves upon the utility-related operating costs observed in Case III, electricity price, feedstock cost, and financial parameters continue to dominate the variability of the methanol MSP. It is also worth noting that differences in utility OPEX across all cases and feedstocks primarily reflect variations in electricity consumption, depending on the presence or absence of PV integration, as further illustrated in Supporting Data by the energy profiles when PV is integrated in Case II by Figures S1 and S2 and for Case IV by Figures S3 and S4.

To contextualize the techno-economic performance of the proposed configurations, the obtained MSP values were benchmarked against other methanol production pathways reported in the literature, including fossil-based methanol synthesis, biomethanol routes, and CO_2_-based e-methanol systems. The MSP values obtained in this study range from 0.684–0.743 €/kg for Cases I–II, while the configurations including auxiliary methanation and hydrogen production (Cases III–IV) show higher values of 1.07–1.24 €/kg due to the substantial electricity demand of electrolytic hydrogen production and the associated capital investment in renewable power infrastructure.

For comparison, current market prices of methanol vary regionally. According to Methanex contract prices for 2026, methanol prices are approximately 0.535 €/kg in Europe, 0.994 $/kg in North America, 0.365 $/kg in Asia, and 0.340 $/kg in China, corresponding roughly to 0.34–0.94 €/kg depending on the market region.^73^ These values indicate that the MSP obtained for the base configurations of this study (Cases I–II) falls within the range of current global methanol prices, whereas the more electrified configurations (Cases III–IV) remain above current market levels. Several studies have reported production costs for alternative methanol synthesis routes. Fossil-based methanol production costs were historically around 0.2 $/kg, but increased significantly during the energy crisis, reaching approximately 2.2 $/kg in Europe in 2022 due to high natural gas prices.^74^ For biobased methanol pathways, IRENA (2021) estimated production costs in the range of 0.320–0.770 $/kg, with potential reductions to 0.220–0.560 $/kg depending on feedstock cost and process improvements.^75^ Similarly, Rinaldi et al. reported a levelized cost of methanol between 0.357 and 0.378 €/kg for a biogas-to-methanol process based on steam reforming.^74^ In contrast, methanol production pathways relying on CO_2_ hydrogenation and electrolytic hydrogen generally exhibit significantly higher production costs due to the electricity demand associated with water electrolysis. For example, Gelten et al. reported production costs between 4059 and 4500 €/t for CO_2_-to-methanol processes depending on the hydrogen supply strategy.^76^ Similarly, Cho et al. estimated a production cost of 1.02 $/kg for a direct CO_2_-to-methanol process using renewable hydrogen.^77^

Future cost projections suggest that e-methanol production could become increasingly competitive as renewable electricity prices decline. Fasihi et al. estimated that global e-methanol production costs may decrease from 1200–1500 €/t in 2020 to approximately 315–350 €/t by 2050 under favorable renewable electricity conditions.^78^ Overall, this comparison indicates that the base biomass-to-methanol configurations (Cases I–II) presented in this study are economically competitive with several reported renewable methanol pathways and fall within the range of current global methanol prices. However, configurations involving extensive electrification and hydrogen production (Cases III–IV) remain more costly due to the high capital and electricity requirements associated with electrolytic hydrogen generation. These results highlight the importance of electricity price, renewable power integration, and hydrogen production costs in determining the economic viability of future methanol synthesis routes.

## Conclusions

A fully electrified, solar-assisted biomass-to-methanol chain was modeled, optimized, and economically assessed for two Spanish biomasses, lignocellulosic and agri-food, within a single integration framework that couples renewable heat and power, advanced CO_2_ management, and waste heat recovery. Electrification of the gasifier and tar reformer eliminates syngas combustion losses, increasing carbon utilization efficiency to 61.6% for the lignocellulosic route and 52.6% for the agri-food route, directly improving methanol yield. Extending the chain with electrolytic H_2_ and captured CO_2_ converts residual carbon to CH_4_, raising total carbon recovery to 82.9 and 68.4% for the lignocellulosic route and the agri-food route, respectively, and further to 87.3 and 73.6% under solar-assisted operation. PV supply cuts grid imports and stabilizes operating costs, delivering the lowest MSPs, 0.708 €/kg for lignocellulosic and 0.785 €/kg for agri-food, while sensitivity analysis confirms that feedstock price dominates MSP once electricity risk is hedged by PV. On the investment side, CAPEX is anchored by the methanol loop and reforming, with the PV + electrolyzer block as the main incremental lever. The agri-food route requires additional drying energy, resulting in higher utility consumption and increased capital investment. Taken together, electrified gasification, renewable-powered utilities, and CO_2_-to-CH_4_ valorization provide a coherent, technically feasible, and highly carbon-efficient route to renewable methanol. Although the integration of the PV system and methanation unit increased both CAPEX and OPEX, it significantly improved the overall carbon conversion efficiency of the process. A comparison with alternative methanol production pathways indicates that electrified biomass gasification combined with renewable hydrogen integration offers a promising approach for improving carbon utilization while maintaining moderate production costs relative to fully hydrogen-based CO_2_ conversion routes. Overall, the results highlight the potential of integrating biomass conversion with renewable electricity to develop flexible Power-to-X systems for sustainable fuel production. However, further work is required to evaluate the environmental performance of the proposed configurations through detailed life-cycle assessment and to assess the impact of large-scale renewable electricity integration on system operation.

## Supplementary Material



## Notes

Methanol synthesis rate expressions, data tables for detailed mass flow rates of cases included and CAPEX, and figures of PV and electricity balance profiles (PDF)

## Nomenclature

**Abbreviations**
BFBG: bubbling fluidized bed gasifier
CAPEX: capital expenditures
CO_2_: carbon dioxide
DEPG: dimethyl ethers of poly­(ethylene glycol)
DB: dry biomass
el: electrical
GJ: giga Joule
GCC: grand Composite Curves
HHV: high Heating Value
HT-WGS: high temperature water gas shift
kW: kilowatt
km: kilometer
MW: megawatt
MeOH: methanol
MER: minimum energy requirement
MSP: minimum selling price
MILP: mixed-integer linear programming
NRTL: non-random two-liquid
OPEX: operating expenditures
op: operating time (hours)
PC-SAFT: perturbed-chain statistical associating fluid theory
PV: photovoltaic
RWGS: reverse water-gas shift reaction
SCG: spent coffee ground
SN: stoichiometric number
Th: thermal
k€: thousand euro
T: tonne
Cu: copper
ZnO: zinc oxide
Al_2_O_3_: alumina
**Symbols**
*Q*: cooling or heating flowrate by utility system (kW)
$: dollar
*K*: equilibrium constant (-)
€: euro
*R*: heat flowrate (kW)
*R*: intervals
*f*_ω_: load factor (-)
*m*: mass flowrate (kg/h)
*N*: number of temperature intervals
*N*_ω_: number of utility units
*y*_ω_: presence or absence of utility system
C: price of feedstock (€/kg), electricity consumed (€/kWh), or revenues from marketable products and byproducts
*W*: net produced power by the utility systems, chemical processes or exchanged with the grid (kW)
*R*: scaling factor
ω: utility unit
*V*: volumetric flowrate (m^3^/h)
