# Supplementary material for: Assessing Biomass-Based Methanol Production via Electrified Gasification and Solar-Assisted CO2 Utilization
Source: Ind Eng Chem Res. 2026 Jun 1;65(22):11595–617. doi: 10.1021/acs.iecr.6c00217 (PMC13262690; doi:10.1021/acs.iecr.6c00217)
Supplement: Supplementary file 1 [file ie6c00217_si_001.pdf]

Supporting Information

Assessing Biomass-Based Methanol Production via Electrified Gasification and Solar-Assisted CO_2 Utilization

*Usman Khan Jadoon[a,b], Pullah Bhatnagar[b], Daniel Flórez-Orrego[b, *], Meire Ellen Gorete Ribeiro Domingos[b], Manuel Rodríguez[a, **], François Maréchal[b]*

[a]Departamento de Ingeniería Química Industrial y del Medioambiente, Escuela Técnica Superior de Ingenieros Industriales, Universidad Politécnica de Madrid, 28006 Madrid, Spain

[b]Industrial Process and Energy Systems Engineering, Ecole Polytechnique Fédérale de Lausanne, EPFL, Sion, 1950, Switzerland

**Corresponding author:* daniel.florezorrego@epfl.ch

***Corresponding author:* manuel.rodriguezh@upm.es

S1. Methanol synthesis rate expression

The generalized rate expression for the Methanol synthesis follows the form as given in Eq.(1).

$$r = \left(\frac{kinetic\, factor \cdot driving\, force}{adsorption\, term}\right) \tag{1}$$

Where the kinetic factor is expressed through an Arrhenius-type correlation given in Eq.(2).

$$k = k_0 T^n exp\left(\frac{-E_a}{RT}\right) \tag{2}$$

The required data for the kinetic factor is included in the Table S1 while the driving force constants are given in Table S2[1]. In addition the concentration exponents and adsorption constants are given in the study by[2]. Eqs. (7) to (9) are given in the main text.

Table S1: Kinetic factors for the rate equations.

Reaction	k	n	E_a[kJ/kmolK]
Eq.(7)	4.0638×10^{-6}[mol^2/g$_{cat}$ sPa]	0	11,695
Eq.(8)	9.0421×10^{8}[mol^2/g$_{cat}$ sPa$^{1/2}$]	0	112,860
Eq.(9)	1.5188×10^{-33}[mol^2/g$_{cat}$ sPa]	0	266,010

Table S2: Driving force constants for reaction.

	Term 1				Term 2			
Reaction	A	B	C	D	A	B	C	D
Eq.(7)	-23.20	14225	0	0	28.895	2385	0	0
Eq.(8)	-22.48	9777	0	0	-28.12	2385	0	0
Eq.(9)	-22.48	9777	0	0	23.974	15,062	0	0

Table S3: Detailed component mass balance for Lignocellulosic biomass to methanol synthesis (Cases I–II)

	Gasification		Reforming	HT-WGSR	CO_2 absorption		MeOH synthesis			Power plant
	Input	Output	Output	Output	Syngas	Pure CO_2	MeOH	Water	Purge	Flue gas
P (bar)	1	1	1	30	30	1	1	1	2	1
T (º C)	30	800	800	362	25	30	30	30	50	120
Component mass flowrate (kg h^{-1})										
Feedstock	16666	-	-	-	-	-	-	-	-	-
water	15000	15637	8512.67	390.27	0	16.15	15.73	63.49	0.08	2145.28
H_2	-	418.57	1674.85	1846.18	1832.55	13.62	0	0	91.47	0.00
N_2	-	38.43	38.43	38.43	37.93	0.49	0	0	37.88	2497.16
O_2	-	0.00	0	0	0	0	0	0	0	1918.23
CO	-	3501	14596.30	12215.70	11976.70	238.95	0	0	65.66	0.00
CO_2	-	4489	4489.97	8230.33	522.06	7465	47.33	0	280.97	2020.22
CH_4	-	707.37	565.90	565.90	536.19	29.68	0.63	0	535.38	0
C_6H_6	-	3234.75	32.35	32.35	0	2.10	0	0	0	0
$C_{10}H_8$	-	1172.60	1.17	1.17	0	0	0	0	0	0
C_6H_6O	-	0.00	0	0	0	0	0	0	0	0
C_2H_4	-	777.83	77.78	77.78	9.54	66.28	0.44	0	9.1	0
Selexol	-	0	0	0	0.02	0.35	0	0	0	0
MeOH	-	0	0	0	0	0	13659.20	6.24	101.05	0

Table S4: Detailed component mass balance for Lignocellulosic biomass to methanol synthesis (Cases III)

	Gasification		Reforming	HT-WGSR	CO_2 absorption		MeOH synthesis			Power plant	Electrolyzer	Methanation
	Input	Output	Output	Output	Syngas	Pure CO_2	MeOH	Water	Purge	Flue gas	Output	Output
P (bar)	1	1	1	30	30	1	1	1	2	1	1	1
T (º C)	30	800	800	362	25	30	30	30	50	120	80	40
Component mass flowrate (kg h^{-1})												
Feedstock	16666	-	-	-	-	-	-	-	-	-	-	-
water	15000	15637	8512.67	390.27	0.00	16.15	15.73	63.49	0.08	2145.28	0	9596.56
H_2	-	418.57	1674.85	1846.18	1832.55	13.62	0	0	91.47	0.00	2132	0
N_2	-	38.43	38.43	38.43	37.93	0.49	0	0	37.88	2497.16	0	0
O_2	-	0	0	0	0	0	0	0	0	1918.23	16833	0
CO	-	3501.00	14596.30	12215.70	11976.70	238.95	0	0	65.66	0.00	0	0
CO_2	-	4489.00	4489.97	8230.33	522.06	7465.00	47.33	0	280.97	2020.22	0	0
CH_4	-	707.37	565.90	565.90	536.19	29.68	0.63	0	535.38	0	0	4265.14
C_6H_6	-	3234.75	32.35	32.35	0	2.10	0	0	0	0	0	0
$C_{10}H_8$	-	1172.60	1.17	1.17	0	0	0	0	0	0	0	0
C_6H_6O	-	0	0	0	0	0	0	0	0	0	0	0
C_2H_4	-	777.83	77.78	77.78	9.54	66.28	0.44	0	9.10	0	0	0
Selexol	-	0	0	0	0.02	0.35	0	0	0	0	0	0
MeOH	-	0	0	0	0	0	13659.20	6.24	0	0	0	0

Note: In Case III, the electrolyzer and methanator operate seasonally (March–October). During November–February, the captured CO_2 is sent to be stored rather than converted through methanation.

Table S5: Detailed component mass balance for Lignocellulosic biomass to methanol synthesis (Cases IV)

	Gasification		Reforming	HT-WGSR	CO_2 absorption		MeOH synthesis			Power plant	Electrolyzer	Methanation
	Input	Output	Output	Output	Syngas	Pure CO_2	MeOH	Water	Purge	Flue gas	Output	Output
P (bar)	1	1	1	30	30	1	1	1	2	1	1	1
T (º C)	30	800	800	362	25	30	30	30	50	120	80	40
Component mass flowrate (kg h^{-1})												
Feedstock	16666	-	-	-	-	-	-	-	-	-	-	-
water	15000	15637.00	8512.67	390.27	0	16.15	15.73	63.49	0.08	2145.28	0	6441.44
H_2	-	418.57	1674.85	1846.18	1832.55	13.62	0	0	91.47	0	1431	0
N_2	-	38.43	38.43	38.43	37.93	0.49	0	0	37.88	2497.16	0	0
O_2	-	0	0	0	0	0	0	0	0	1918.23	11298	0
CO	-	3501.00	14596.30	12215.70	11976.70	238.95	0	0	65.66	0	0	0
CO_2	-	4489.00	4489.97	8230.33	522.06	7465.00	47.33	0	280.97	2020.22	0	0
CH_4	-	707.37	565.90	565.90	536.19	29.68	0.63	0	535.38	0	0	2862.86
C_6H_6	-	3234.75	32.35	32.35	0	2.10	0	0	0	0	0	0
$C_{10}H_8$	-	1172.60	1.17	1.17	0	0	0	0	0	0	0	0
C_6H_6O	-	0	0	0	0	0	0	0	0	0	0	0
C_2H_4	-	777.83	77.78	77.78	9.54	66.28	0.44	0	9.10	0	0	0
Selexol	-	0	0	0	0.02	0.35	0	0	0	0	0	0
MeOH	-	0	0	0	0	0	13659.20	6.24	101.05	0	0	0

Table S6: Detailed component mass balance for food-waste-to-methanol synthesis (Cases I–II)

	Gasification		Reforming	HT-WGSR	CO_2 absorption		MeOH synthesis			Dryer
	Input	Output	Output	Output	Syngas	Pure CO_2	MeOH	Water	Purge	Flue gas
P (bar)	1	1	1	30	30	1	1	1	2	1
T (º C)	30	800	800	354	25	30	30	30	50	120
Component mass flowrate (kg h^{-1})										
Feedstock	16666	-	-	-	-	-	-	-	-	-
water	15000	15647	8563.00	259.42	0.00	8.60	3.54	52.81	0.02	8648.50
H_2	-	406.71	1645.31	1781.80	1781.71	0	0	0	70.80	0
N_2	-	291.47	291.47	291.47	287.05	4.00	0.04	0	287.00	36718.20
O_2	-	0	0	0	0	0	0	0	0	7791.60
CO	-	2962.91	13975.99	12079.00	11538.83	0	0	0	81.90	0
CO_2	-	3148.49	3148.49	6131.39	181.84	6052.61	20.76	0.00	115.24	1199.50
CH_4	-	472.59	378.07	378.07	371.86	6.22	0.58	0.00	356.53	0
C_6H_6	-	3234.75	32.35	32.35	0	0	0	0	0	0
$C_{10}H_8$	-	1172.61	1.17	1.17	0	0	0	0	0	0
C_6H_6O	-	0	0	0	5.82	0	0	0	0	0
C_2H_4	-	777.91	77.79	77.79	55.50	9.28	0.32	0	5.50	0
Selexol	-	0	0	0	0.01	0.29	0	0	0	0
MeOH	-	0	0	0	0	0.00	13288.60	15.19	127.54	0

Table S7: Detailed component mass balance for food-waste-to-methanol synthesis (Cases III)

	Gasification		Reforming	HT-WGSR	CO_2 absorption		MeOH synthesis			Dryer	Electrolyzer	Methanator
	Input	Output	Output	Output	Syngas	Pure CO_2	MeOH	Water	Purge	Flue gas	Output	Output
P (bar)	1	1	1	30	30	1	1	1	2	1	1	1
T (º C)	30	800	800	354	25	30	30	30	50	120	80	40
Component mass flowrate (kg h^{-1})												
Feedstock	16666	-	-	-	-	-	-	-	-	-	-	-
water	15000	15647	8563.00	259.42	0	8.60	3.54	52.81	0.02	8648.50	0	7411.82
H_2	-	406.71	1645.31	1781.80	1781.71	0	0	0	70.8	0	1340	0
N_2	-	291.47	291.47	291.47	287.05	4.00	0.04	0	287	36718.2	0	0
O_2	-	0	0	0	0	0	0	0	0	7791.6	13028	0
CO	-	2962.91	13975.99	12079.00	11538.83	0	0	0	81.9	0	0	0
CO_2	-	3148.49	3148.49	6131.39	181.84	6052.61	20.76	0	115.24	1199.5	0	0
CH_4	-	472.59	378.07	378.07	371.86	6.22	0.58	0	356.53	0	0	3294.14
C_6H_6	-	3234.75	32.35	32.35	0	0	0	0	0	0	0	0
$C_{10}H_8$	-	1172.61	1.17	1.17	0	0	0	0	0	0	0	0
C_6H_6O	-	0	0	0	5.82	0	0	0	0	0	0	0
C_2H_4	-	777.91	77.79	77.79	55.50	9.28	0.32	0	5.50	0	0	0
Selexol	-	0	0	0	0.01	0.29	0	0	0	0	0	0
MeOH	-	0	0	0	0	0	13288.6	15.19	127.54	0	0	0

Note: In Case III, the electrolyzer and methanator operate seasonally (March–October). During November–February, the captured CO_2 is sent to be stored rather than converted through methanation.

Table S8: Detailed component mass balance for food-waste-to-methanol synthesis (Cases IV)

	Gasification		Reforming	HT-WGS		CO_2 absorption		MeOH synthesis			Dryer	Electrolyzer	Methanation
	Input	Output	Output	Input	Output	Syngas	Pure CO_2	MeOH	Water	Purge	Flue gas	Output	Output
P (bar)	1	1	1	30	30	30	1	1	1	2	1	1	1
T (º C)	30	800	800	300	354	25	30	30	30	50	120	80	40
Component mass flowrate (kg h^{-1})													
Feedstock	16666	-	-	-	-	-	-	-	-	-	-	-	-
water	15000	15647	8563.00	1480.92	259.42	0	8.60	3.54	52.81	0.02	8648.50	0	6030.94
H_2	-	406.71	1645.31	1645.31	1781.80	1781.71	0	0	0	70.80	0.00	1340	0
N_2	-	291.47	291.47	291.47	291.47	287.05	4.00	0.04	0	287.00	36718.20	0	0
O_2	-	0	0	0	0	0	0	0	0	0.00	7791.60	10601	0
CO	-	2962.91	13975.99	13975.99	12079	11538.83	0	0.00	0	81.90	0	0	0
CO_2	-	3148.49	3148.49	3148.47	6131.39	181.84	6052.61	20.76	0	115.24	1199.50	0	0
CH_4	-	472.59	378.07	378.07	378.07	371.86	6.22	0.58	0	356.53	0.00	0	2680.42
C_6H_6	-	3234.75	32.35	32.35	32.35	0	0	0	0	0	0	0	0
$C_{10}H_8$	-	1172.61	1.17	1.17	1.17	0	0	0	0	0	0	0	0
C_6H_6O	-	0	0	0	0	5.82	0	0	0	0	0	0	0
C_2H_4	-	777.91	77.79	77.79	77.79	55.50	9.28	0.32	0	5.50	0	0	0
Selexol	-	0	0	0	0	0.01	0.29	0	0	0	0	0	0
MeOH	-	0	0	0	0	0	0	13288.60	15.19	127.54	0	0	0

Table S9: Cost of system components used for CAPEX and OPEX

	Base Investment Cost	Scaling factor	Base scale	Installation factor	Maximum Size	Ref.
			Pretreatment			
Conveyors	0.2 M€$_{2001}$	0.8	16.66 ton h^{-1}	1.86	110	3
Storage	4.34 M€$_{2001}$	0.65	16.66 ton h^{-1}	1.86	110	3
Grinding	0.27 M€$_{2001}$	0.27	16.66 ton h^{-1}	1.86	110	3
Dryer	0.63 M€$_{2001}$	0.8	16.66 ton h^{-1}	1.86	110	3
Metal removal	0.227 M€$_{2001}$	0.7	16.66 ton h^{-1}	1.86	110	3
Feeding	0.204 M€$_{2001}$	1	16.66 ton h^{-1}	1.86	110	3
			Gasifier			
BFBG	6.042 M€$_{2001}$	0.65	16.66 ton h^{-1}	1.69	83	3
Cyclone	0.089 M€$_{2001}$	0.7	36 m^3 gas/sec	2.8	200	3
			Gas cleaning			
Tar cracker	2.86 M€$_{2001}$	0.7	33.85 m^3 gas/sec	1.86	52	3
HT-WGSR	2.079 M€$_{2001}$	0.85	15.6 $MW_{LHV,db}$	1	-	3
			CO_2 capturing			
Selexol	2.52 M€$_{2001}$	0.7	139.247 kmol CO_2 h^{-1}	1	-	3
			MeOH synthesis			
MeOH	2.087 M€$_{2001}$	0.6	13.313 ton h^{-1}	2.1	-	3
Refining	3.728 M€$_{2001}$	0.7	13.313 ton h^{-1}	2.1	-	3
			Methanation			
Methanator	0.995 M€$_{2014}$	1	39.762 MW_{SNG}	1	175	4
Catalyst	0.191 M€$_{2014}$	1	39.762 MW_{SNG}	1	175	4
Electrolyser	303 €kW^{-1}	1	73.711 MW_{el}	1	-	5

Note: The cost of solid wate handling specifically the ash is assumed from the study of biomass gasification plant, which is 75.58 €/ton and landfill taxes 114.65 €/ton [6]. The cost of equipment like pumps, heat exchangers, compressors, pressure vessels was calculated by the methodology given by [7].

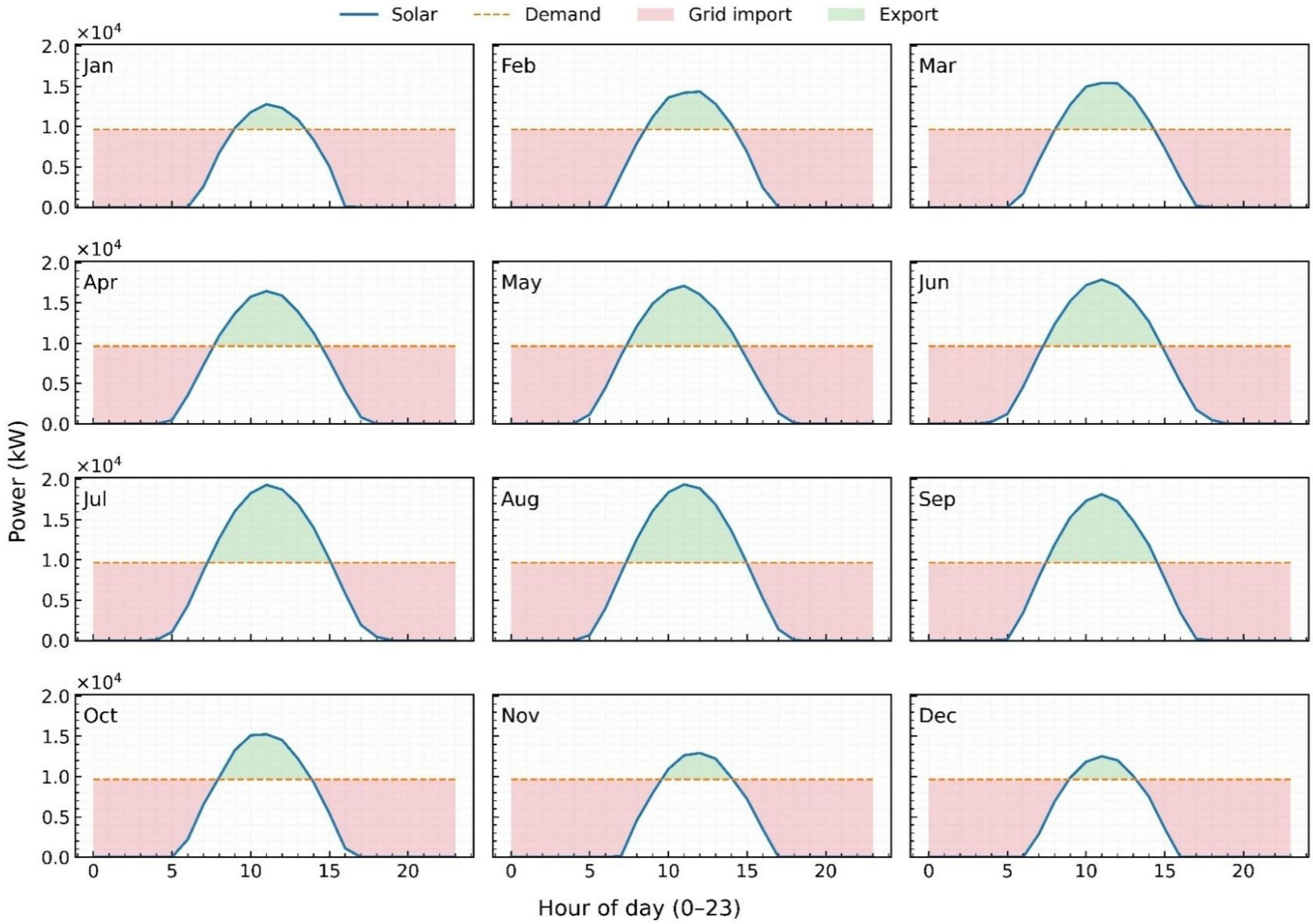

Figure S1 PV profiles in the case II for Lignocellulosic-based route.

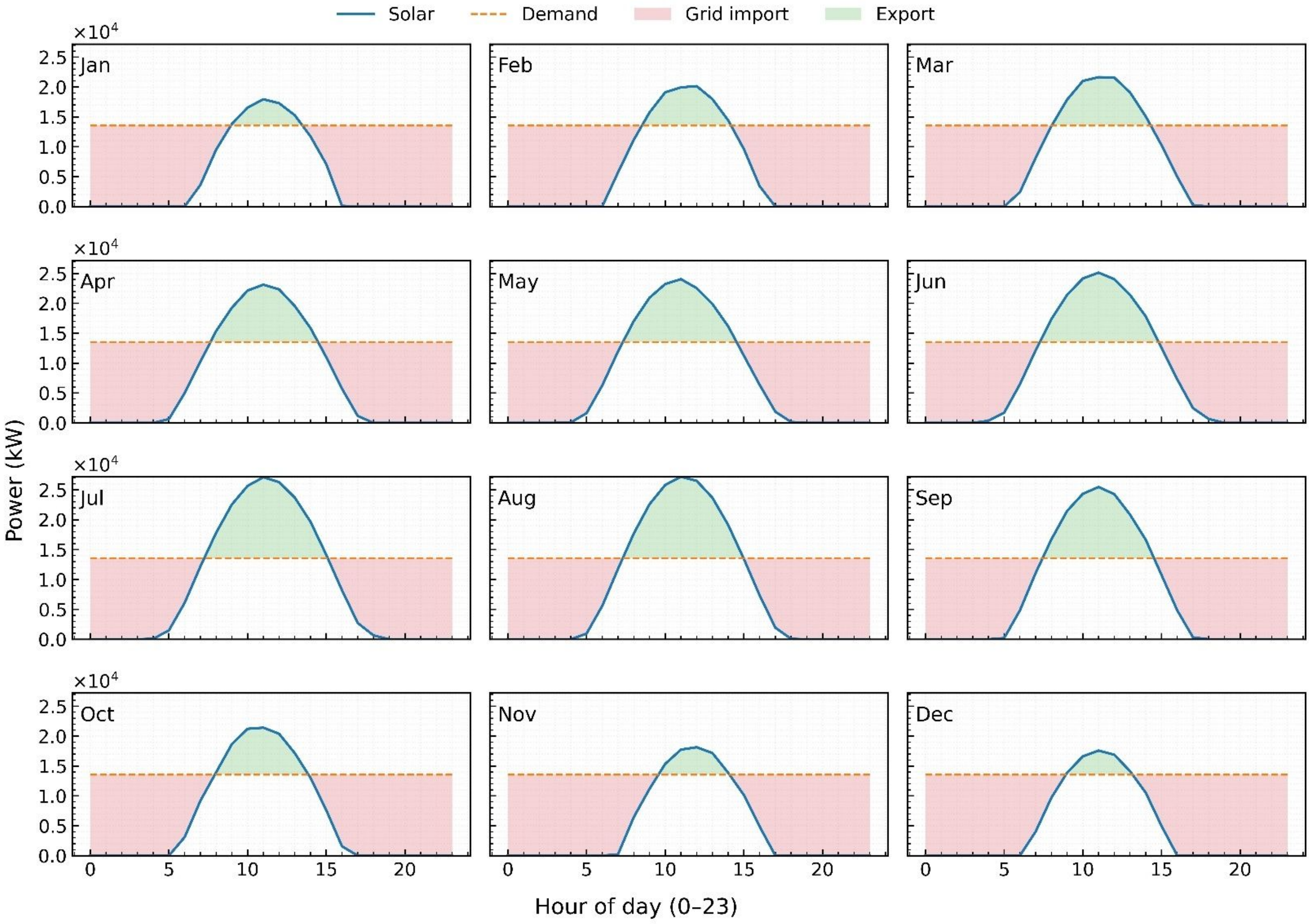

Figure S2 PV profiles in case II for agri-food based route.

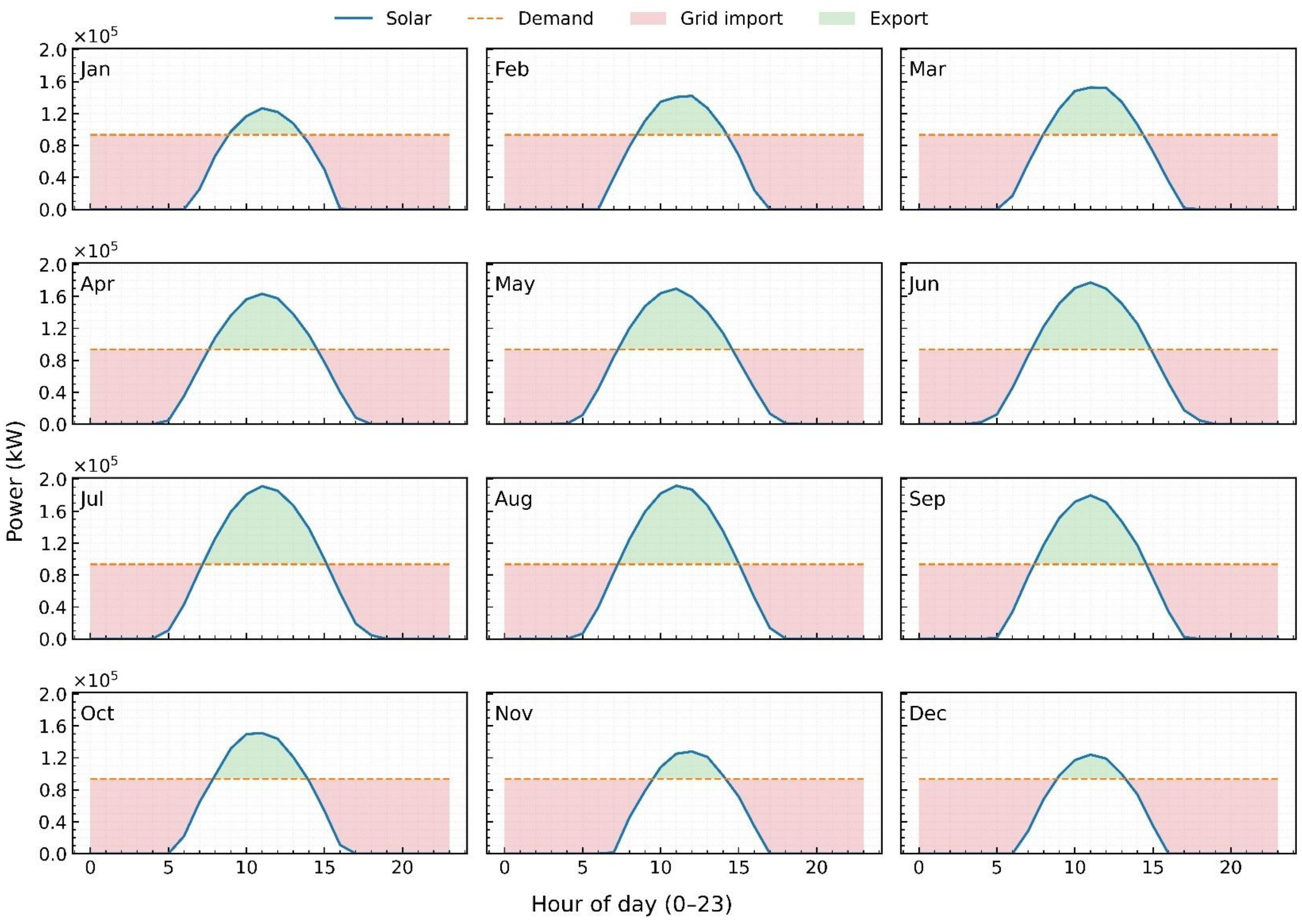

Figure S3 PV profiles in case IV for Lignocellulosic-based route.

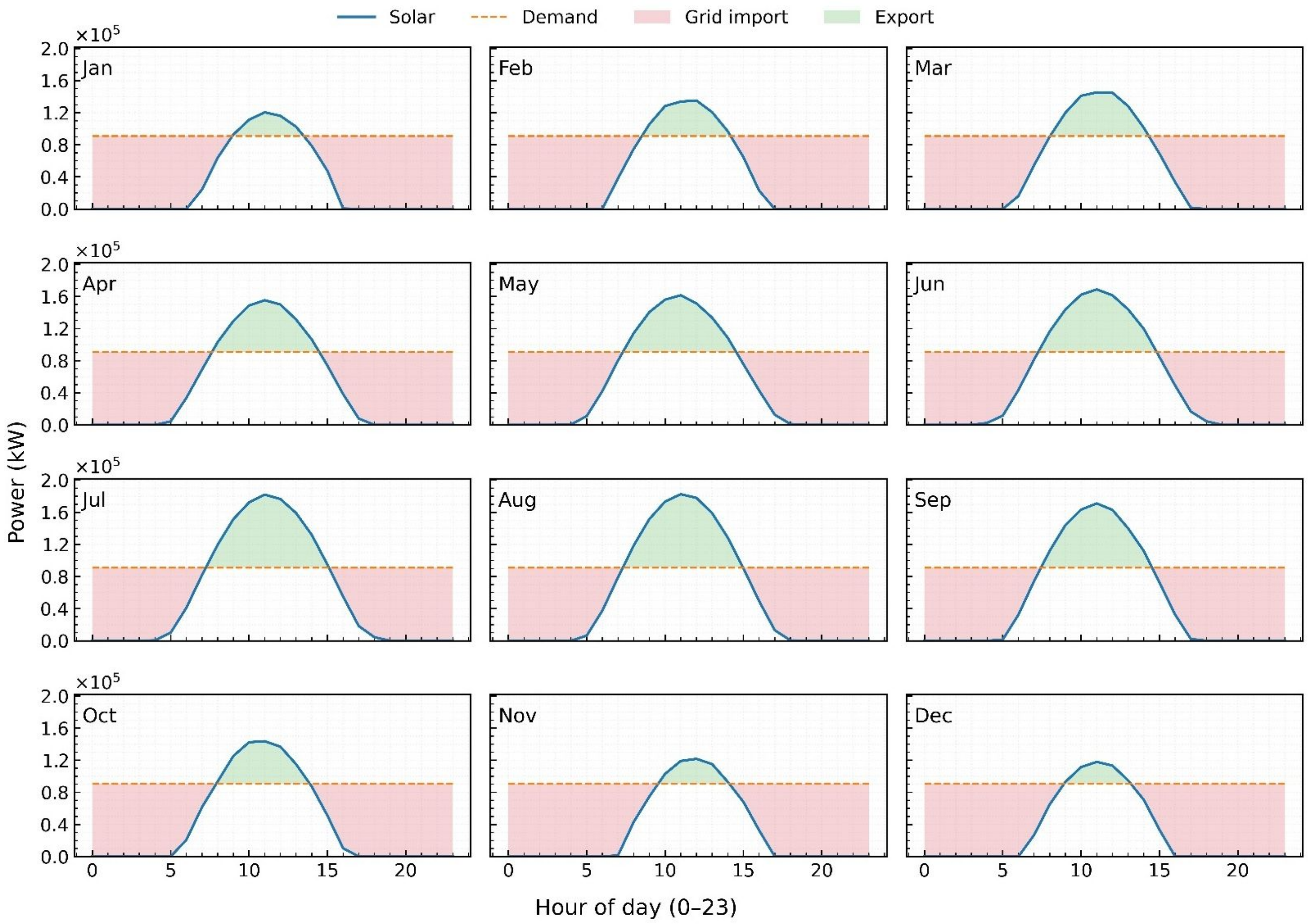

Figure S4 PV profiles in Case IV for agri-food based route.

Reference:

(1) Lim, H.-W.; Park, M.-J.; Kang, S.-H.; Chae, H.-J.; Bae, J. W.; Jun, K.-W. Modeling of the Kinetics for Methanol Synthesis using Cu/ZnO/Al2O3/ZrO2 Catalyst: Influence of Carbon Dioxide during Hydrogenation. *Industrial & Engineering Chemistry Research* **2009**, *48* (23), 10448-10455. DOI: 10.1021/ie901081f. An, X.; Zuo, Y.; Zhang, Q.; Wang, J. Methanol Synthesis from CO2 Hydrogenation with a Cu/Zn/Al/Zr Fibrous Catalyst. *Chin J Chem Eng* **2009**, *17* (1), 88-94, Article. DOI: 10.1016/S1004-9541(09)60038-0 Scopus.

(2) Kiss, A. A.; Pragt, J. J.; Vos, H. J.; Bargeman, G.; de Groot, M. T. Novel efficient process for methanol synthesis by CO2 hydrogenation. *Chemical Engineering Journal* **2016**, *284*, 260-269. DOI: https://doi.org/10.1016/j.cej.2015.08.101.

(3) Hamelinck, C. N.; Faaij, A. P. Future prospects for production of methanol and hydrogen from biomass. *Journal of Power sources* **2002**, *111* (1), 1-22.

(4) Holmgren, K. Investment cost estimates for gasification-based biofuel production systems. IVL Svenska Miljöinstitutet: 2015.

(5) Lichner, C. Electrolyzer prices–what to expect. *pv magazine International. Accessed: September* **2024**, *11*. Rahmat, Y.; Maier, S.; Moser, F.; Raab, M.; Hoffmann, C.; Repke, J.-U.; Dietrich, R.-U. Techno-economic and exergy analysis of e-methanol production under fixed operating conditions in Germany. *Applied Energy* **2023**, *351*, 121738.

(6) Bastien, É. J. M. Towards Circular Economy: Wood ash management for biomass CHP plants in the UK. 2020.

(7) Turton, R.; Bailie, R. C.; Whiting, W. B.; Shaeiwitz, J. A. *Analysis, synthesis and design of chemical processes*; Pearson Education, 2008.
